# Cost-effectiveness of community-based gendered advisory services to farmers: Analysis in Mozambique and Tanzania

**DOI:** 10.1371/journal.pone.0211448

**Published:** 2019-03-20

**Authors:** Tewodaj Mogues, Valerie Mueller, Florence Kondylis

**Affiliations:** 1 Fiscal Affairs Department, International Monetary Fund, Washington, DC, United States of America; 2 Development Strategy and Governance Division, International Food Policy Research Institute, Washington, DC, United States of America; 3 Development Research Department, World Bank, Washington, DC, United States of America; University of Georgia, UNITED STATES

## Abstract

Rigorous impact evaluations on agricultural interventions in the developing world have proliferated in research of recent years. Whereas increased care in causal identification in such analyses is beneficial and has improved the quality of research in this field, much of the literature still fails to investigate the costs needed to achieve any benefits identified. Such understanding, however, would be crucial for drawing policy and programmatic conclusions from the research and for informing the allocation of public investments. Cost-effectiveness analysis (CEA) subjects both the cost side and the effects side of agricultural and rural interventions to technical scrutiny and unifies both sides in order to compare the relative cost-effectiveness of different modalities of a programme, of efforts to reach different target groups, or of efforts to achieve different outcomes. CEAs, while present in the health and education sectors, remain rare in agricultural and rural development research. This study contributes to filling the knowledge gap by conducting CEAs in a particular type of programmatic work in the agricultural sector—namely, interventions conducted as field experiments that bring a gender lens to community-based advisory services in African rural areas. Specifically, we consider two such programmes—one in Mozambique in which such advisory services aim to improve sustainable land management (SLM) practices in agricultural production, and the other in Tanzania to advise farmers on their land rights. Using CEA methods combined with econometric analysis based on randomised controlled trials, we find that the gendered modality is consistently more cost-effective than the basic modality when considering varied outcomes and target groups. However, for any given modality, it is more cost-effective to improve outcomes for men than for women. The structure of costs in the agricultural extension programme further allowed for a simulation of how cost-effectiveness would change if the programme were scaled up geographically. The results show that expansion of the basic modality of the SLM programme leads to improvements in cost-effectiveness, while the gendered modality displays nonlinear changes in cost-effectiveness along the expansion path, first worsening with initial scale-up and subsequently improving with further expansion.

## 1. Introduction

### 1.1. Motivation

Impact evaluations have taken agricultural development research by storm, becoming more common in absolute terms as well as relatively more prevalent within the development literature. Rigour in causally identifying the impact of agricultural interventions and programmes on farmers’ productivity and welfare, among other outcomes, has also increasingly been able to influence the direction of public investments made by international agencies and developing-country governments in support of the agricultural sector and rural areas. For example, in the World Bank, there were more projects in the agriculture and rural development sector that included rigorous impact evaluations, compared to all other sectors; and the share of all World Bank agriculture projects that conducted impact evaluations grew rapidly over time [1].

But academic impact evaluations in agricultural development, however careful in identifying the cause-and-effect relationship between agricultural interventions and development outcomes, have mostly been silent on the costs required to achieve the benefits. Systematic reviews of the presence of cost-effectiveness or other careful cost analysis of agricultural impact evaluations do not exist to the best of our knowledge. The closest work that comes to this is a study focusing on a subcategory within agriculture, namely public-sector incentives to farmers to protect biodiversity on farmland. This review finds that of 2,000 such studies, fewer than 5 per cent include any meaningful cost data pertaining to these public initiatives [2].

Arguably, the cost side is an important aspect especially in cases where such research seeks to inform policies and investments on the ground. In a few topics, such as agricultural input subsidies, attention has been paid to the cost of agricultural initiatives in comparison to the size of the benefits these same expenditures could have brought about had they been invested in alternative areas. But even in the case of input subsidies, examination of the cost side has with a few exceptions (for example, [3]) not been subjected to the same care and rigour as the analysis of the benefit side of agricultural programmes in standard impact evaluations. Cost-effectiveness analysis in development that subjects both the costs and effects of programmes to equal technical scrutiny, and brings them together in order to compare alternative interventions or modalities within interventions, is rare in the agricultural sector. In contrast, it has been far more common in the health and education sectors (see [4–7]).

It is at this point worth methodologically to contrast cost-effectiveness analysis (CEA) with two other approaches that centrally bring in the cost aspects when evaluating programmes, policies or interventions. One approach is cost-benefit analysis (CBA). The central difference to CEA is that it expresses not only the costs but also the benefits of the programmes in monetary value terms. (Table A in S1 Appendix summarises some key differences between CEA and CBA concise form). CBA may be appropriate in many contexts. For example, as applied to agriculture, studies have compared the costs with the economic profitability or value of alternative ways of disposing of residues from rice farming in France [8], of the application of biochar as a soil amendment technology in two regions of the world [9], or of nonprice export promotion policies in the US [10]. These examples also illustrate the diversity in empirical methods used to carry out CBA, ranging from using statistics from meta-analyses of the literature [9], to collecting interviewed key-informants’ expert assessments on costs and the value of benefits [8], to simulation analysis [10].

A second approach focuses on the economic and social returns to public expenditures on different sectors such as agriculture, infrastructure or health (see [11] for a review of this body of work). Most studies in this area employ rigorous econometric analysis of the impact of public expenditures on various outcomes. Empirically, these may be country-level estimations drawing on subnationally-disaggregated or household level data, or cross-country panel data. This line of analysis may or may not capture benefits in monetary terms, depending on which is more appropriate given the research question.

Neither of these two broad approaches—CBA, or returns to spending analysis—would be appropriate for our case at hand. The outcomes of interest in our inquiry are not easily measured in financial terms. While, for example, farmers’ increased knowledge about their land rights can ultimately be hypothesised to carry economic value, for example through increased private investment in their land upon a better understanding of their property rights, these are further downstream effects than we are directly seeking to measure. Therefore, CBA as an approach does not lend itself well to our study.

Secondly, while the returns-to-expenditures approach does not need to denote benefits in monetary terms, it applies best to larger-scale public expenditure policies, enabling a comparison, for example, of the agricultural productivity returns to increasing public spending on agriculture versus on road infrastructure. In contrast, we are here interested in specific interventions and programmes related to land use and land rights, rather than the sweep of total expenditures in agriculture. Therefore, in considering the contribution of our study, we focus on its value-addition to the CEA literature relating to agriculture, the large gaps of which we laid out above.

Our analysis presents CEAs on a prominent area in the agricultural sector, namely advisory services to farmers. Specifically, it provides guidance on how scarce public resources can best be allocated to achieve improved knowledge, attitudes, and practices on land rights for farmers and on agricultural management of land, through paralegal aid and agricultural extension interventions, respectively. The regional settings are Mozambique and Tanzania. Both interventions make use of trained and skilled community members to provide advisory services to farmers, and give special attention to reaching women and men with those services. These programs were implemented as part of separate randomised controlled trials, which each collected a rich set of household, individual, and cost survey data. We provide detailed discussion of the CEA methodology employed in these advisory services contexts, including results based on differing assumptions and simulation under scale-up scenarios. While this article, as empirical analysis, is contextually situated by the nature of the interventions examined, we argue, in light of the significant gap in the literature described, that our analysis can serve as a useful framework for conducting future CEAs in agricultural development.

The next subsection lays out the programmatic context, describing the advisory services to Mozambican and Tanzanian farmers. Section 2 discusses the empirical method used. The data are described in Section 3, followed by the results of the CEAs in Mozambique and Tanzania. The final section concludes.

### 1.2. The community-based, gendered advisory services in Mozambique and Tanzania

The primary concern of the two interventions studied here is the delivery of advisory services, with particular attention to reaching both women and men smallholders. The importance of providing agriculture-relevant advice to both men and women farmers in developing countries emanates not only from equity concerns, but has emerged from numerous findings on the overall productivity- and welfare-increasing effect of doing so for farm households. Research has shown that joint agricultural decisionmaking between men and women in the same household improves conditions for the household as a whole, when compared to decisionmaking only by the man. Examples from the subregion of our empirical setting substantiate this: jointly managed plots in Kenya are more likely to employ sustainable land management practices and improved seeds in Kenya [12] and greater soil fertility application in Mozambique [13], and joint decisionmaking on tree management led to high-density planting in Malawi [14]. Advisory services that reach women can give them a stronger foundation for engaging in such decisions and enabling the realisation of such benefits for the household.

The general modality employed in the programmes in this study is to train qualified members of the community in both technical and soft skills so that they can be the conduit of rural advisory services to other farmers in their community. The programme in Mozambique focused on providing advice on appropriate sustainable land management (SLM) practices in agricultural production, while the Tanzanian intervention reached out to farmers with advice and information on land property rights, so that awareness of those rights would ultimately reduce land-related conflict and improve farmers’ investment in their land as a consequence of strengthened tenure security. The implementation of both programmes took place in the form of randomised controlled trials, with randomisation at the community level. Thus, our impact evaluation in this study is able to employ an experimental approach. While our study integrates results from impact evaluation with costs, to produce a cost-effectiveness analysis, recent studies have carried out more detailed impact evaluations (without cost analysis) of the Mozambique programme ([15], [16]) and of the Tanzania intervention ([17]).

The next two subsections provide an overview of the two programmes. Further details on the design and coverage of the programmes are captured in Sections 2 and 3, respectively.

#### 1.2.1. Advisory services in agricultural production techniques

The agricultural production advisory services programme took place in five districts across three provinces of Mozambique from 2010 to 2013, under the auspices of a broader World Bank smallholder development project in the country’s Zambezi valley [18]. Unless explicitly stated otherwise, this article focuses all discussion of the Mozambique activities on the advisory services programme, rather than the broader World Bank smallholder project, which included a number of additional agricultural activities without a gender focus and without following an experimental design.

The programme’s primary objective was to improve smallholders’ knowledge and adoption of appropriate SLM practices in agricultural production. Its approach toward that objective relied on the use of contact-farmers from within the communities as conveyers of SLM messages to other farmers. Within this general approach, the programme employed two primary types of delivery of extension services, with a gender feature constituting the primary distinction between the two.

In the first modality, one farmer in each community was assigned to serve as the contact-farmer for all other farmers in the community. The contact-farmers received training on SLM practices as well as equipment and tools to enable them to have their plot (or one of them, if there were multiple) function as a demonstration plot. Communities usually already had a person functioning as contact-farmer—usually male—prior to the project, and the project worked with those individuals. In the second modality, in addition to the preexisting male contact-farmer, an additional female contact-farmer was identified, and she received the same training and tools as the male contact-farmer. The objective of this modality was to improve access by women farmers to information and extension about agricultural conservation practices, under the expectation that women contact-farmers are, for cultural reasons and due to the often gendered nature of social interaction in rural societies of many developing countries, better positioned to convey conservation messages to women.

#### 1.2.2. Advisory services on farmers’ land rights

The second rural advisory services project focused on the provision of community-based legal aid to farmers to improve their knowledge about land rights and about the role of government agencies in shaping and protecting such rights. It was implemented from May 2013 through July 2014 by a Tanzanian nongovernmental organisation, Mama’s Hope Organisation for Legal Assistance (MHOLA), in two districts of the Kagera region of Tanzania. Similar to the agricultural extension programme, the community-based legal aid intervention used as its core modality the sourcing of qualified members of the community and the training of those members in the hard and soft skills of land-related advisory service provision. They would then serve as resource persons on land property rights and conflicts, as well as be able to refer farmers to more formal government agencies for adjudication of specific cases when needed.

Like the agricultural extension programme, the land advisory services programme was highly sensitive to the gendered nature of knowledge and attitudes about land rights and the types of demand for advice given on land-related matters. Paralegal workers were trained, among other, on the differences in the bundles of rights that women and men have with respect to land held by the household, as well as the different informal norms that govern claims women and men can make on land under various circumstances. Unlike the agricultural extension programme, the land legal aid initiative was not implemented through two different modalities with respect to sensitivity to gender.

## 2. Materials and methods

The main datasets on intervention costs did not involve human subjects. The institutional review board (IRB) of the International Food Policy Research Institute (IFPRI) and the Tanzania Commission for Science and Technology approved the collection and management process of the data used in this study that involves human subjects in Tanzania (i.e. the household and individual surveys). Informed consent was oral, given that the respondents were mostly nonliterate. However, the oral consent was obtained accompanied by a documented signature by the respondent. The IRB approved this informed consent procedure. For the Mozambique surveys, institutional review board approval was not required as it was the government of Mozambique that was formally responsible for collecting the data, and not the research team.

Before describing the data used in analysis of the cost-effectiveness of these rural advisory services, we first provide in detail the methodology employed in identifying and computing the relevant costs and their components, describe the use of information on programme coverage in scaling the effects of the programme, and present the analysis of the programme impact as a key ingredient in the denominator of cost-effectiveness ratios. The method is both laid out in general terms as well as tailored to some of the specificities of the two advisory services interventions.

### 2.1. Accounting for costs

The neglect of two important features in other cost frameworks often leads to nontrivial underestimation of costs. First, the actual (rather than budgeted) costs spent on staff of the implementing agency, which are only obtainable with time sheets and other adequate instruments. Second, the costs borne not only by the implementation agency but by other organizations upstream. The nature of public expenditures involved in program implementation is crucial to simulate the implications of expanding or replicating the intervention. Accounting for public costs outside of the programme required greater attention in the Tanzania case, where it was imperative to collect monthly data on the time cost of public officials who were not project staff yet provided their services to the programme. Such services included participation in workshops to train paralegals, meetings, and other undertakings in connection with the interventions.

Programmes usually involve activities, and therefore costs, at multiple levels, or tiers—for example, costs that accrue at the community level within a country, the regional level, and the central level. Improperly accounting for costs accruing at different tiers may lead to under- or overstating the costs of interventions. In the analysis, we categorise costs by tier. Total costs specific to a tier are defined here as those that would increase only in the event of inclusion of an additional such tier but do not change with treatment of additional units below this tier. For example, total administrative post costs in the Mozambique programme—such as the payment of salary of the two extension agents—will increase only through inclusion of a post into the project. But administrative post costs do not increase if additional communities within the same post are inducted into the project; what does increase in the latter scenario are community-level costs only. The same logic applies to tier-specific costs at the other levels.

Conceptually, we generalize tier-specific costs at seven levels, corresponding with the Mozambique case, in increasing hierarchical level: farmers, communities, localities, administrative posts (or just ‘posts’), districts, provinces, and the central level. Tier-specific costs are denoted as *e*. For example, the tier-specific cost at the post level, and in particular for the *p*^th^ post, is *e*_*p*_. The number of posts in total is *P*. Then, the totality of tier-specific costs for posts is $\sum_{p=1}^{P}e_{p}$. Using analogous notation for all other tiers—that is, *f*, *m*, *l*, *d*, *v*, and *n* for farmers, communities, localities, districts, provinces, and the central level, respectively—total project costs *C* are:

$$C=e_{n}+\sum_{v=1}^{V}e_{v}+\sum_{d=1}^{D}e_{d}+\sum_{p=1}^{P}e_{p}+\sum_{l=1}^{L}e_{l}+\sum_{m=1}^{M}e_{m}+\sum_{f=1}^{F}e_{f}$$ (1)

### 2.2. Periodicity

Planning of the broader Mozambique initiative that contains the project under analysis, i.e. CGAAS, began as early as 2007 [18]. However, the study project with its specific intervention characteristics was not operationalised until 2010, and it was concluded in 2013. The CGLAS programme spanned the period of May 2013 through July 2014. The time dimensions of the CGAAS and CGLAS programmes feature in the cost analysis in four primary ways. First, the periodicity is accounted for explicitly in the way that capital costs are annualised. Execution of the programmes entails expenditures of different durability. Public works and construction of infrastructure imply larger bulk costs that generate outputs that remain usable over a number of years, while operational costs and services are provided on a continuous basis throughout the project period. We therefore annualise capital costs by spreading the cost of each capital item from the time of acquisition over the useful life of the asset. In order to annualise capital costs, we first need to estimate the useful lives for different capital items. Absent information on this that is specific to Mozambique and to Tanzania, we estimate these conservatively as follows: For the Tanzania capital items, we were able to find similar corresponding items, along with their useful lives, in the US government guidelines for property and equipment capitalisation of the different types of capital goods (Section 1.35.6.10, “Property and Equipment Capitalisation,” in the *Internal Revenue Manual* of the Internal Revenue Service). We double these useful lives in our cost data, assuming that capital goods in Tanzania will be in active use for at least double the time of those in developed countries like the United States. In Mozambique, the vast share of capital costs emerges from the construction of village housing for the extension agents at their work sites. Given that there is not a good analogue of this in the abovementioned U.S. Internal Revenue Manual, we assume a useful life of 10 years for these village based structures, and more generally for all capital costs in Mozambique.

To implement this, we first classify costs at each tier *a* and each time period *t* into capital (*k*) and recurrent (*b*) expenditures, that is, $e_{at}=e_{at}^{k}+e_{at}^{b}$. We then annualise the cost of each asset *A*_*as*_ accruing at tier *a* and acquired at time period *s*, and obtain capital expenditures $e_{at}^{k}$ by summing over the period from asset acquisition to the time period *t—*that is,

$$e_{at}^{k}=\sum_{s=1}^{t}\frac{r∙A_{as}}{1-\frac{1}{{\left(1+r\right)}^{T_{Aas}}}},$$ (2)

where *T*_*Aas*_ is the estimated useful life of asset *A*_*as*_ and *r* is the interest rate. In other words, capital expenditures at a time period *t* include the annualised cost of all capital assets acquired for the project in that year and before.

Second, all costs are converted from nominal (i.e. current) to real (i.e. constant) values. We use the annual consumer price index (CPI) for Mozambique from the World Development Indicators database, and the monthly CPI for Tanzania from the country’s National Bureau of Statistics. The base year for both is 2010.

Third, present values are derived to the time of the project start for each cost item in period *t*.

Fourth and finally, as described later in Section 3.4, the surveys for both initiatives were conducted in two rounds, in the form of a midline survey and an endline survey for CGAAS, and with a base- and endline survey for CGLAS. We therefore conduct the CEA both at midline and at endline in the former, and just at endline in the latter case.

These second to fourth aspects of periodicity lead to this formulation for tier-specific costs at endline:

$$e_{a}^{end}=\sum_{t=1}^{T_{end}}\left[\frac{100}{{CPI}_{t}}\frac{e_{at}}{{\left(1+r\right)}^{t}}\right]$$ (3)

where *CPI*_*t*_ is the consumer price index for each period and *T*_*end*_ is the period of the endline survey. The expression is analogous for costs at the time of the midline survey in the case of CGAAS.

### 2.3. Disaggregation by programme modality

As remarked earlier, CEA is in most cases appropriately used to conduct comparisons in cost-effectiveness rather than to evaluate the cost-effectiveness of a programme in absolute terms. In Section 1.2.1 we described the two distinct modalities employed in carrying out CGAAS—one modality had a focus on ensuring gender balance among the contact-farmers, while the other did not. In this vein, one of the key comparisons this study undertakes is between the gendered and the basic extension modalities. Eq (1) represents total programme costs, but now we seek to separately determine costs of each modality. Communities are the units of treatment and the modalities are thus carried out in mutually exclusive communities. Thus, the last two of the seven summands of Eq (1) are distinct by treatment modality, while the other five are not. The total costs associated with each intervention type *j* then are:

$$C^{j}=\alpha^{j}∙(e_{n}+\sum_{v=1}^{V}e_{v}+\sum_{d=1}^{D}e_{d}+\sum_{p=1}^{P}e_{p}+\sum_{l=1}^{L}e_{l})+\sum_{m=1}^{M^{j}}e_{m}+\sum_{f=1}^{F^{j}}e_{f}$$ (4)

where *j* = {*g*, *ng*, *ns*} may either represent the gendered (*g*) or the nongendered (*ng*) i.e. basic treatment subject to this study, or the activities of the broader World Bank smallholder project that are not subject (*ns*) to the impact- and cost-effectiveness evaluation (the distinction between the broader project and the evaluated interventions was discussed in Section 1.2.1). This notation is introduced since some, especially higher tier cost items are available only aggregated across all activities (*g*, *ng*, *ns*) and our empirical approach includes estimates of the shares of these cost items that are associated with the treatments of interest (superscripted *g* and *ng*). The shares assigned to each type of treatment/activity add up to one, that is, *α*^*g*^ + *α*^*ng*^ + *α*^*ns*^ = 1. From this it follows that *C*^*g*^ + *C*^*ng*^ + *C*^*ns*^ = *C*; *M*^*g*^ + *M*^*ng*^ + *M*^*ns*^ = *M*; and *F*^*g*^ + *F*^*ng*^ + *F*^*ns*^ = *F*. The final cost-effectiveness results only account for the cost components of interest—i.e. *C*^*g*^ and *C*^*ng*^—and do not include those cost components (*C*^*ns*^) that pertain to activities not evaluated.

### 2.4. Scale-up analysis

A challenge that academic work needs to rise to is providing more analytical insights on how a programme, often executed and thus evaluated on a narrow scale, would perform if it were scaled up. Our paper concerns itself with this question. To simulate the evolution of cost-effectiveness of CGAAS and its components, we first need to define precisely what form of scaling up will be assessed. In our analysis, the intervention is said to have been scaled up from communities to localities if all localities that contain at least one community originally receiving the intervention are, after scale-up, “saturated” with the intervention.

To demonstrate how expenditures change in the process of scaling up, by way of example suppose one of the intervention modalities *j* originally operates over a particular geographic space; then it expands so as to saturate localities. After scale-up the total expenditure on the treatment type *j* is

$$C_{ul}^{j}=\alpha_{ul}^{j}∙(e_{n}+\sum_{v=1}^{V}e_{v}+\sum_{d=1}^{D}e_{d}+\sum_{p=1}^{P}e_{p}+\sum_{l=1}^{L}e_{l})+\sum_{m=1}^{M_{ul}^{j}}e_{m}+\sum_{f=1}^{F_{ul}^{j}}e_{f}.$$ (5)

The subscript *u* signifies that the expression pertains to the scenario after an upscaling or expansion of the intervention type *j*, and the subscript *l* similarly indicates that the nature of the scale-up is one that leads to a saturation of localities, that is, by including additional communities into the project within those localities that originally contained one or more intervention communities. Comparing the elements in Eqs 4 and 5, it is clear that $\alpha_{ul}^{j}\geq \alpha^{j}$ given that the share of communities that fall under intervention type *j* will naturally increase after expansion, that is, given that $M_{ul}^{j}\geq M^{j}$. The same obtains with the number of farmers exposed to the treatment. However, in an expansion through saturation of localities, all costs at the post and higher levels remain unchanged, even though the share of them attributable to the intervention type does increase.

It is useful to point out that there is a key difference in the characteristics of costs disaggregated by intervention modality *j*, between the case of the original analysis and a scale-up scenario. In the latter, we are simulating a scaling up of the programme types one at a time—that is, first we consider scaling up, for example, the gendered treatment, and in a separate scenario consider the scale-up of the basic treatment. That is because both in our analytical approach, as well as in our data, we have different cost structures under each modality, and there are a multitude of ways that a simultaneous scale-up of both the basic and the gendered intervention modality would proceed (for example, in the case of a post that originally includes both gendered and basic treatment communities, which of the two treatment types would an originally untreated community in that post receive?). For this reason, we consider the more unambiguous scenarios of scaling up one modality at a time.

### 2.5. Effect aggregation

#### 2.5.1. Coverage information for aggregating the effects

So far, the methodological details outlined have focused on deriving the appropriate cost quantities. CEA needs, however, to also explicitly account for the coverage of the programme in order to ultimately normalise costs by the programme’s reach. Analysis of the intervention draws on information on the total number of individuals by intervention community who are potentially affected by the programme, disaggregated by groups relevant to the analysis. And in the case of the CGAAS programme, which is geographically highly staggered compared with CGLAS, we also draw on details regarding the jurisdictional coverage of the programme—that is, the number of intervention communities in each locality, the number of such localities in each post, and so forth.

The Mozambique and Tanzania interventions targeted individuals with extension and land rights messages, respectively. In both programmes, the individuals targeted were household heads as well as spouses of heads. The impact evaluation (described below) also focuses on deriving the impact of the initiatives on household heads and their spouses. Therefore, the relevant population in the coverage consists of such heads and heads’ spouses. Gendered analysis is key in this study, and therefore the coverage data of this population of heads and heads’ spouses are disaggregated by gender. Since, as detailed above, CEA in Mozambique will be comparative between the gendered and the basic modalities of the programme, the coverage data are further disaggregated by these farmers subjected to the gendered and the basic extension treatments.

#### 2.5.2. Regression analysis to derive effects from the experimental interventions

We bring together normalised costs and the relevant coefficients of the impact evaluation to generate the cost-effective ratios (CERs). The impact evaluations of both interventions use an experimental approach, with random assignment at the community level (see Section 3.4 for further details on the randomisation and the survey data collection). Data were collected on a range of outcomes and other characteristics on both the household head and his or her spouse (if existent) were captured in a midline survey in Mozambique and a baseline survey in Tanzania, and then again in an endline survey in both (see Section 3.4 for sample size details). In Mozambique, the same survey was additionally administered to all contact-farmers.

The impact evaluation coefficients used in the CERs of this study derive from the regression

$$Y_{fhm}=\beta +\gamma_{q}B_{m}+X_{fhm}^{\prime }\delta +\varepsilon_{fhm},$$ (6)

where Y indicates an outcome of interest with regard to individual/farmer *f* in household *h* and community *m*; *B* is a dummy variable equal to 1 if the community is subjected to the intervention and 0 if it is a control community, and thus the coefficient of primary interest is *γ;* and, finally, *X* is a vector capturing a host of control variables (household and individual demographic variables, land size, housing characteristics, etc.). Standard errors are clustered at the community level.

The results are obtained with six main variations. The first type of variation consists of the outcomes of relevance to the study. Of interest is to understand how the programmes affect farmers’ awareness, knowledge, and adoption of practices supported by the intervention—these are SLM practices in CGAAS and rights and rules with regard to farmland property in CGLAS. The second type of variation pertains only to the Mozambique programme, which has two different treatment arms: *B* may represent the gendered or the basic treatment, or may pool both as a general extension treatment. Third, the analysis is separately conducted to examine the effect on women and on men. In the case of CGAAS, it is also conducted for just the contact-farmers. Fourth (again only in CGAAS) the impact is derived both at the time of the midline survey and at the time of the endline survey.

Fifth, in the case of the Tanzania programme, communal structures offer opportunities for meetings, including those organised by the paralegal worker, that may span across communities. This suggests including an analysis that accounts for potential spillovers to nearby communities. Sixth and finally, given that by the time of the endline survey in the CGLAS programme only 84 per cent of the paralegal workers still resided in the communities they were serving, we carry out a treatment-impact-on-the-treated (TOT) estimation in addition to the intent-to-treat (ITT) analysis.

### 2.6. Costs and scaled effects as cost-effectiveness ratios

To finally derive the CERs, total cost by treatment type is normalised by its coverage and divided by the impact coefficient, so that

$${CER}_{G,t}^{j}=\frac{C_{t}^{j}}{\gamma_{qGt}^{j}∙N_{qGt}^{j}}$$ (7)

for intervention type *j* (gendered treatment, basic treatment, or pooled treatment) and consideration of key (that is, heads and spouses of heads) farmers of gender *G* (male, female, or pooled across genders), where *N* signifies the number of farmers under these treatment- and gender-categories. The full set of subscripts and superscripts, not always indicated previously for cleaner notation, are shown here to emphasise that the CER will vary by treatment type and time point of evaluation in the case of Mozambique, by method of impact evaluation (intent-to-treat, treatment impact on treated, and accounting for potential spillovers) in the case of Tanzania, and by gender of beneficiaries for both programmes.

Note that the method of estimation (reflected by subscript *q*) changes not only the impact coefficient γ but also the assumed coverage of treatment *N*. For example, in the case of the ITT estimation, all communities originally assigned to treatment are considered for the population count, but in the case of the TOT estimation, it is the communities actually treated that are considered in *N*. Importantly, CERs are derived only in cases where there is an impact γ that is statistically significantly different from zero, as it is only sensible to identify the cost-to-effectiveness ratio if there is in fact a statistically nonzero effect. Thus, all results presented meet this condition. Nonetheless, we present the level of statistical significance in each case. Section A in S1 Appendix shows all impact coefficients (including the statistically insignificant ones) along with their associated standard errors.

## 3. Results

### 3.1. Description of the cost data

For each of the two programmes, we collected detailed data on costs incurred by the implementation agency for the period of the project—May 2013 through July 2014 for CGLAS and 2010 to 2013 for CGAAS. The data were recorded monthly in Tanzania, and in an even more detailed manner in Mozambique, by time of acquisition or expenditure of each item or activity. The costs were all based on actual expenditures, and not on budgeted figures. In the case of CGLAS, cost data were submitted by MHOLA to the research team every three months, while in the case of CGAAS data were obtained from the project after project completion. All data were available highly disaggregated by labour cost (including type of labour and type of compensation such as allowances, salary, stipends, and so forth), transport costs (for example, fuel), capital outlays (such as purchase of motorcycles and furniture), office operational costs, workshop and training expenses, and other categories.

In the Tanzania project, expenditures were recorded at the district level of the two intervention districts, such that the intervention cost can be measured separately for each district. Some types of costs that were more centralised and were not direct field outlays were assigned to the central project office in Bukoba. The spatial details of costs are richer and more complex in the Mozambique programme. Costs at the lowest (community) level centre around goods and services directly provided to contact-farmers. Those contact-farmers are trained on agricultural conservation practices as well as provided the necessary equipment and agricultural inputs to enable them to use some of their land as a demonstration plot. Contact-farmers also receive bicycles so that they can travel more easily to individual farmers and advise them on SLM techniques directly at the site of those farmers’ land. This aside, contact-farmers do not receive a salary for their time commitment. Among the extension methods to convey SLM practices to farmers is the use of so-called farmer-field-days (events in which farmers attend a plot of a host contact farmer who showcases his or her new farming practices), which incurs costs, for example, to reach farmers in the community with the announcement of the field-days in order to ensure strong attendance.

The most significant costs at the administrative post level are those associated directly with extension officers, of which two are deployed in each post. Extension officers receive salary, and living quarters are constructed for them and their family. Each is equipped with a motorcycle to reach contact-farmers throughout the intervention communities in their post. At the district level, the programme put in place a district facilitator and an environmental specialist to attend to various aspects of the project. Those two staff are also responsible for providing an intensive training for the extension officers on SLM techniques. The district facilitators and environmental specialists are given a vehicle for their co-ordination and training work in their district. Finally, central costs include all the expenses of compensation of non-field-project staff for them to design and conduct the programme, their travel costs to get to the field sites, capital outlays such as for vehicles, and various operational expenditures.

In CGAAS, the empirical results are obtained across the board for four degrees of inclusiveness of different levels of costs (this not not similarly applicable in CGLAS, given the much lower complexity of spatial aspects of its cost structure). With cost-inclusiveness, we are referring to the extent to which costs at higher tiers, as described above, are accounted for (versus are left out) in the cost-effectiveness analysis. For example, the highest degree of inclusiveness accounts for all four cost tiers, that is, for costs from the community to the central levels. The next highest degree of inclusiveness accounts for costs at the community, post, and district levels, leaving out central project costs. The remaining two more narrowly defined scopes for cost-inclusiveness follow analogously. Where post, district, and central project costs are accounted for, these are “distributed” across the intervention areas, in accordance with the share of communities under each intervention type. For example, in the analysis of cost-effectiveness of the gendered treatment arm, the share of central project costs included is equal to the share of all communities that are subjected to this treatment arm (see also the earlier Section 2.1).

### 3.2. Descriptive summary of programme costs

Tables 1 and 2 as well as Table Y in S1 Appendix present disaggregations of the total cost of the projects under evaluation. Over the evaluation period of 2010 to 2013 of the Mozambique programme, total project costs amounted to about 109 million Mozambican meticais (MZN for short), or US$3.7 million—at the exchange rate of US$1 to 29.6 MZN prevailing in mid-2013—of which the largest share, 42 per cent (46 million MZN), was incurred at the district level (see Table Y) (all subsequent references to $ pertain to US$). Central expenditures, such as on overall project design and consultations with the relevant government and other actors in Mozambique, were also substantial, about a quarter of all costs. Expenditures closer to the ground, such as to hire and deploy extension agents (post level) and to train and provide contact-farmers with the requisite equipment (community level), were relatively smaller. The bulk of spending was incurred in the middle years of the evaluation period.

**Table 1:** CGAAS: Summary of the agricultural services programme costs, by location and category.

Cost categories	Sofala province		Tete province	Zambezia province		Central level	All tiers
	Chemba district	Maringue district	Mutarara district	Mopeia district	Morrumbala district		
Capital	649	3,116	4,978	3,088	2,946	2,090	16,868
Goods	1,698	815	2,743	2,195	3,470	819	11,740
Labour	1,807	1,807	1,269	1,172	1,172	18,054	25,283
Training	821	1,415	1,850	1,017	1,937	922	7,961
Operating costs	8,461	4,247	13,869	6,677	9,287	5,042	47,583
All categories	13,436	11,401	24,709	14,149	18,813	26,927	109,436

Notes: CGAAS = community-based gendered agricultural advisory services. Values in 1,000 Mozambican meticais. Exchange rate prevailing in mid-2013: US$1 = 29.6 MZN.

**Table 2:** CGLAS: Summary of costs of the paralegal programme, by year, location, and category.

Cost categories	2013			2014			All tiers and periods
	Biharamulo district	Karagwe district	Central level	Biharamulo district	Karagwe district	Central level	
Labour	8,544	9,334	11,058	5,890	5,890	7,697	48,413
Capital	0	350	5,300	0	0	0	5,650
Space rental	300	300	417	700	700	0	2,417
Training	34,550	34,181	1,100	0	0	0	69,832
Transport	135	100	1,140	0	0	1,428	2,803
Stationery	200	200	885	700	700	770	3,455
Meetings, other services	1,130	1,082	1,446	188	557	775	5,177
Food, other goods	8,226	7,872	0	0	0	0	16,098
All categories	53,085	53,420	21,346	7,478	7,847	10,669	153,845

Notes: CGLAS = community-based gendered land advisory services. Values in 1,000 Tanzanian shillings. Exchange rate prevailing in mid-2014: US$1 = 1,626 TZS.

As Table 1 shows, operating costs make up a significant part of the programme’s expenditures, amounting to about 44 per cent of expenditures. The second largest expense type is personnel compensation. Public works—for example, construction of housing for extension agents—make up the third largest category. Training for technical staff, extension agents, and contact-farmers is in fact the smallest expenditure type in amount. Costs by district range from about $388,000 to $840,000, but central-level costs exceed those of the highest-cost district at about $916,000 (note that the costs by district reported in Table 1 include all costs at the community, post, and district level associated with each district, and thus are larger than the district-level cost row in Table Y).

Table 2 presents an overview of CGLAS. By far the largest cost components are training expenditures, as well as salary and other personnel compensation. The programme costs were frontloaded, into 2013, given that many of the preparatory activities take place at the early stages of the project, such as training the paralegals and acquiring vehicles and other capital items. In the aggregate, district-level costs are fairly evenly distributed between the two programme districts. At 21 per cent, central expenditures make up a fairly modest share of all costs.

### 3.3. Programme coverage

Table 3 shows the coverage of CGAAS, including the total number of men and women exposed to each treatment arm (details on the assignment of communities to treatment are given in the next subsection). This coverage description excludes communities that were not part of the impact evaluation area—and in which the World Bank supported programme conducted other interventions different from the treatments described in Section 1.2.1. In other words, it does not include areas in which activities were carried out that correspond to the cost component subscripted with *ns* in Eq (4).

**Table 3:** CGAAS: Coverage of the agricultural advisory services programme in Mozambique.

Coverage		Sofala province		Tete province	Zambezia province		All locations
		Chemba district	Maringue district	Mutarara district	Mopeia district	Morrumbala district	
Posts		3	3	4	2	4	16
Localities		6	5	14	8	14	47
Localities involving basic treatment		6	3	10	7	13	39
Localities involving gendered treatment		5	4	9	7	7	32
Localities involving either treatment		6	4	11	8	13	42
Communities under a single treatment		15	15	15	15	15	75
Communities under either treatment		30	30	30	30	30	150
Individuals exposed to basic treatment	*Men*	1,265	1,184	5,481	8,679	5,026	21,635
	*Women*	1,164	1,128	5,038	7,872	4,575	19,776
	*All*	2,429	2,312	10,519	16,551	9,601	41,412
Individuals exposed to gendered treatment	*Men*	1,297	1,616	14,962	5,860	8,359	32,095
	*Women*	1,207	1,543	13,610	5,390	7,576	29,326
	*All*	2,504	3,159	28,572	11,250	15,935	61,421
Individuals exposed to any treatment	*Men*	2,562	2,800	20,444	14,539	13,385	53,730
	*Women*	2,371	2,671	18,648	13,262	12,150	49,102
	*All*	4,933	5,471	39,092	27,801	25,536	102,833

Notes: CGAAS = community-based gendered agricultural advisory services. The number of communities under the basic treatment is the same as the number under the gendered treatment in each district. Given that in our project area each treatment arm is present in at least one community of each post, for each district the number of posts involving basic and gendered is identical to the total number of posts.

However, the coverage does capture all—rather than only the survey sampled—households and individuals that were exposed to the treatments. As discussed earlier, exposure is defined by being a household head, or a spouse of a household head, in treatment communities. The numbers of exposed men and women are estimated based on data on the number of household heads and their spouses across the treatment communities from the household surveys, data on those individuals’ gender, and Mozambique population census data on the number of households by community. As the table shows, a total of about 102,800 people were exposed to the project. Of those, somewhat more were men than women, and more farmers were located in areas where the gendered treatment was conducted than the number of farmers under the basic treatment.

Table Z in S1 Appendix presents the simulated coverage of the programme as it goes to scale, under two expansion scenarios: when the programme components are scaled up to saturate localities, and when they are further scaled up to saturate posts. Note that the latter expansion is exactly equivalent to a full scale-up to all areas of the five project districts, given that each intervention type originally takes place in at least one community of each post in the project districts. The expansion scenarios we will analyse by simulation constitute a significant scaling up of the programme: in the locality saturation scenario, the number of farmers under the gendered (basic) treatment increases by a factor of 4.7 (8.4), to more than 287,000 (345,000) farmers. In the post/district-saturation scenario, the number of farmers increases more than sixfold (ninefold) relative to the original programme, to more than 382,000 farmers.

Table 4 presents the coverage of CGLAS. By design, 70 villages were part of the intervention, with an equal number in each district. These villages overall are located within 32 wards. The number of individuals exposed to the treatment, at about 91,000, is somewhat lower than the number in the Mozambique programme (about 103,000), with the number of women somewhat larger than men. The distribution of coverage is distinctly smaller in one district than in the other, given differences in village population size across the two districts.

**Table 4:** CGLAS: Coverage of paralegal land advisory services programme in Tanzania.

Coverage		Karagwe	Biharamulo	Total
Number of wards		18	14	32
Number of communities		35	35	70
Individuals exposed to treatment	*Men*	26,697	16,488	43,185
	*Women*	30,383	17,730	48,113
	*All*	57,080	34,218	91,298

Notes: CGAAS = community-based gendered agricultural advisory services.

### 3.4. Description of the household and individual surveys

In the evaluated CGAAS programme, communities are randomly assigned to the gendered treatment, the basic treatment, or a control status. Districts serve as strata for the randomisation: In each district, 15 communities were randomly assigned into each of the two treatment arms, and another 10 to control status.

From each study community, households were randomly selected to be surveyed. The survey used for the impact evaluation of CGAAS was conducted in two rounds in the form of a midline survey in 2012 and an endline survey in 2013. Eighteen farm households were randomly selected in each of the survey communities and one to two (depending on the treatment arm) contact-farmer households per community were interviewed as well. 75 communities were under each treatment arm and 50 in the control group. This resulted in a target sample size of up to 4,000 households. Individual-specific variables—on which our outcomes primarily rely—were captured from the household head and from the head’s spouse separately. Ultimately, 5,884 individuals were surveyed in the midline survey, and 5,076 in the endline survey.

In CGLAS, communities are randomly assigned to either the (single) treatment or control group. The planned study communities consisted of a census of all 140 rural communities across the two study districts (there were 70 in each). The intervention was randomised across the communities stratified by district, resulting in 70 treatment and 70 control communities, with 35 in each district, respectively. Subsequently, one control district had to be eliminated due to improper consideration of an urban as a rural community, resulting in a total of 139 study communities.

The CGLAS impact evaluation survey data are obtained from a baseline survey conducted in April 2013 and an endline survey in September 2014. Respondents were the household head and the spouse (where existent) of the head from 12 households randomly drawn from each survey community. Given this design, the target sample size amounted to 1,668 households, including each head and spouse per household.The final count of individual respondents was 2,413, of which 1,575 are female and 838 male.

The outcomes of interest in CGAAS are to determine how the SLM extension programme and its gendered and basic modalities affected farmers’ understanding and application of agricultural conservation and other SLM practices. Specifically, we consider farmers’ awareness, knowledge, and adoption of specific practices on which contact-farmers have been trained and supported to on-train other farmers. In total, six SLM techniques are considered. Contour farming is the practice of planting crops along the contours of a sloped plot of land, so that elevation does not vary along a row of crops. This reduces erosion of the soil on land that is not flat. In the practice of strip-tillage, field surface is only minimally disturbed and instead just the specific location where seeds will be inserted are tilled, thus reducing the moisture loss that results from conventional tilling. Pit planting has similarities in purpose with strip tillage: a small pit is dug into which the seed is placed, enabling the farmer to efficiently employ water and fertiliser. Crop rotation prevents the soil nutrient depletion of monoculture, by the planting of diverse types of crops in successive agricultural seasons, as different crops use up soil nutrients in different intensities. Soil conditions and moisture are also conserved through mulching, the practice of covering the land with materials such as organic residues. This also helps to reduce weed growth. Finally, row planting involves growing crops in rows that are spaced widely enough to enable the farmer to better inspect the crops for needed interventions, and to increase access to sunlight for the plants.

The analysis of CGLAS focuses on the ways that the community-based paralegal aid programme affected women’s and men’s perceptions, attitudes, and in some cases actions taken with regard to land rights and procedures. These land rights questions include: rights that men and women should be able to have with regard to land, procedures to seek redress in cases of land disputes, the quality and fairness of the work done by various land administration and adjudication bodies, and their engagement with these bodies and interactions they have sought out with them.

Table 5 describes the working definitions of awareness, knowledge, and adoption of both the SLM techniques as well as the land rights issues, and compares them with the use of these terms in other literature. It is apparent that while our study, like that of others, employs definitions that are suited to the specific context and interventions of interest, we follow a blend between the definitions commonly used in the agricultural extension literature, and those found in Knowledge, Attitudes and Practices (KAP) surveys, which are often employed in the health literature but also found elsewhere such as in land use studies [19].

**Table 5:** Awareness, knowledge and adoption—definitions used in this study and in other literature.

CGAAS	CGLAS	Examples from other studies	
*Awareness and attitudes*, based on survey questions asking…			(combines awareness and knowledge): whether respondent has knowledge of at least one of a list of conservation measures [20]
whether the respondent has come across SLM technique X	respondent’s perception and attitude on land rights issue X	a ‘consciousness question’: whether respondent is perfectly aware of the presence of extension services [21]; respondent’s willingness to join groups involved active on land use management [19]	
*Knowledge*, based on…			
basic test questions pertaining to SLM technique X, then considering a score greater than a technically determined threshold to constitute having good knowledge about the technique. The exams were developed from the training manuals administered to the contact-farmers.	basic test questions pertaining to land rights issue X, then considering a score greater than a technically determined threshold to constitute having good knowledge about the land issue. The exams were developed from the training manuals administered to the community-based paralegal aid workers.	survey questions on who respondent received extension from, what type of extension they received, and what means were used to provide extension [21]; response to request to define land-use and cover change, and evaluation whether response is adequately close to correct definition or not [19]	
*Adoption and practices*, based on survey question…			
on whether the respondent applied SLM technique X in the 12-month period prior to the survey date.	about actions respondent has taken with regard to land rights issue X (time range varies by question).	on whether the respondent used the extension services [21]; whether respondent uses any of a list of conservation measures [20]; whether there was current or previous involvement on the part of the respondent in any land use management programme [19]	

### 3.5. Cost-effectiveness of agricultural advisory services in terms of contact-farmers’ outcomes

Based on Eq (7), Table 6 presents the CERs for the impact of the gendered treatment on contact-farmers’ knowledge of various SLM practices, and knowledge of any SLM practices, referred to as general SLM. (Table 5 had described how knowledge was determined in the survey, and Section 3.4 detailed each technique mentioned in Table 6.) It is critical, at the outset, to restate what we discussed in greater detail in the methodology section—namely, that CERs should never be interpreted as reflecting costs that bring about *only* the impact implied in the particular CER. In this case, the intervention potentially has, of course, impacts on a range of outcomes for the contact-farmers—some of which we present in Table 6—as well as on a range of outcomes for ordinary farmers (covered in Section 3.6).

**Table 6:** CGAAS: Cost-effectiveness of the gendered treatment of the programme in increasing contact-farmers’ SLM knowledge.

SLM techniques	Cost-inclusiveness—costs up to:			
	Central	District	Post	Community
Contour farming	623.82**	483.79**	244.02**	150.96**
Strip tillage	805.03***	624.32***	314.91***	194.80***
Pit planting	1,290.51*	1,000.82*	504.81*	312.28*
Crop rotation	1,341.72*	1,040.54*	524.84*	324.67*
Mulching	4,448.85*	3,450.20*	1,740.27*	1,076.55*
General SLM	1,523.03**	1,181.15**	595.77**	368.55**

Notes: CGAAS = community-based gendered agricultural advisory services; SLM = sustainable land management. Values in 1,000 Mozambican meticais. Exchange rate prevailing in mid-2013: US$1 = 29.6 MZN. Cost-effectiveness ratios are based on costs and impact by the time of the endline survey. CERs are only reported for cases with statistically significant underlying effects (see further details in Section 2.6).

***, **, and * indicate statistical significance at the 1, 5, and 10 per cent levels, respectively.

For example, the CER in the last column and first row of Table 6 shows that (accounting only for community level costs) increasing an additional farmer’s knowledge of contour farming practices costs 151 thousand MZN. But the same project leads to a variety of additional outcomes (seen in the subsequent rows of Table 6, for example). That is, the costs cannot be disaggregated by the different outcomes they produce, and outcomes cannot be aggregated in CEA the way benefits are aggregated in cost–benefit analyses (CBAs) where outcomes are captured in monetary terms, as was discussed in Section 1.1. Rather than evaluating CERs in their absolute magnitude, therefore, one should use them in comparative fashion, to determine the relative cost-effectiveness of different intervention components, of different reaching outcomes, and for targeting different groups of individuals.

The CERs in Table 6 are given for different degrees to which higher-level costs associated with the intervention are accounted for, as discussed earlier. While it is natural to expect that CERs are larger the greater is the extent to which higher-tier costs are accounted for, Table 6 shows how pronouncedly sensitive the results are to such degrees of cost inclusion. Contrasting the two extreme cases—inclusion of only community-level costs (i.e., Community column) versus inclusion of the costs of the intervention at all four tiers of operation (i.e., Central column)—the former is about one-quarter the size of the latter. CERs that ignore only expenses of the intervention at the highest level are somewhat over three-quarters of the CERs based on the full costs, and CERs accounting for the two lowest levels of expenses are about 40% of the CERs based on the full costs.

Table 6 shows that, among the SLM practices, the project was most cost-effective for upgrading contact-farmers’ skills with regard to contour farming practices, followed by strip tillage, and it was least cost-effective in strengthening skills about mulching practices. The CERs differ pronouncedly, with the CERs for increasing contour farming knowledge being less than 15 per cent of the CERs for mulching. A caveat is in order at this stage: In several cases, the confidence intervals around the CERs—based on confidence intervals around the underlying impact coefficients in the denominator—overlap. Thus, the differences between the CERs, especially when these are small in magnitude should be seen as indicative and suggestive.

Whereas the CERs in Table 6 focus only on the gendered treatment arm, results in Table 7 enable a comparison of the cost-effectiveness of the gendered treatment arm with the broader intervention, which includes both gendered and basic treatments. CERs of the gendered treatment are about 58 to 80 per cent of the CERs of the overall intervention. In other words, irrespective of the type of SLM technique, or the extent of costs considered, the gendered treatment appears to be more cost-effective than the programme as a whole.

**Table 7:** CGAAS: Cost-effectiveness of the intervention in increasing SLM awareness.

Cost-inclusiveness	SLM techniques	Gendered treatment	Any treatment
Central	Contour farming	529.96***	774.41**
	Pit planting	662.97**	951.02*
	Row planting	461.90*	803.09***
District	Contour farming	411.00***	593.70**
	Pit planting	514.15**	729.10*
	Row planting	358.22*	615.69***
Post	Contour farming	207.30***	284.28**
	Pit planting	259.33**	349.12*
	Row planting	180.68*	294.81***
Community	Contour farming	128.24***	164.18**
	Pit planting	160.43**	201.63*
	Row planting	111.77*	170.26***

Notes: CGAAS = community-based gendered agricultural advisory services; SLM = sustainable land management. Values in 1,000 Mozambican meticais. Exchange rate prevailing in mid-2013: US$1 = 29.6 MZN. Cost-effectiveness ratios are based on costs and impact by the time of the endline survey. CERs are only reported for cases with statistically significant underlying effects (see further details in Section 2.6).

***, **, and * indicate statistical significance at the 1, 5, and 10 per cent levels, respectively.

The cost-effectiveness of the interventions in increasing contact-farmers’ awareness of SLM approaches can, as before, also be compared across these approaches. The gendered intervention has had associated with it the lowest cost for impact on awareness about row planting techniques, and the highest cost per impact in the context of pit planting as an SLM method. The cost-effectiveness ranking of awareness-raising of SLM techniques is not the same across intervention types considered. For the broad intervention (combining gendered and basic treatments), the greatest cost-effectiveness is achieved for contour farming, but as with the gendered treatment, the least cost-effective are the efforts to increase awareness about the pit planting technique. (This comparison across SLM practices is unaffected by the level inclusiveness of costs underlying the CERs.)

**Fig 1:**
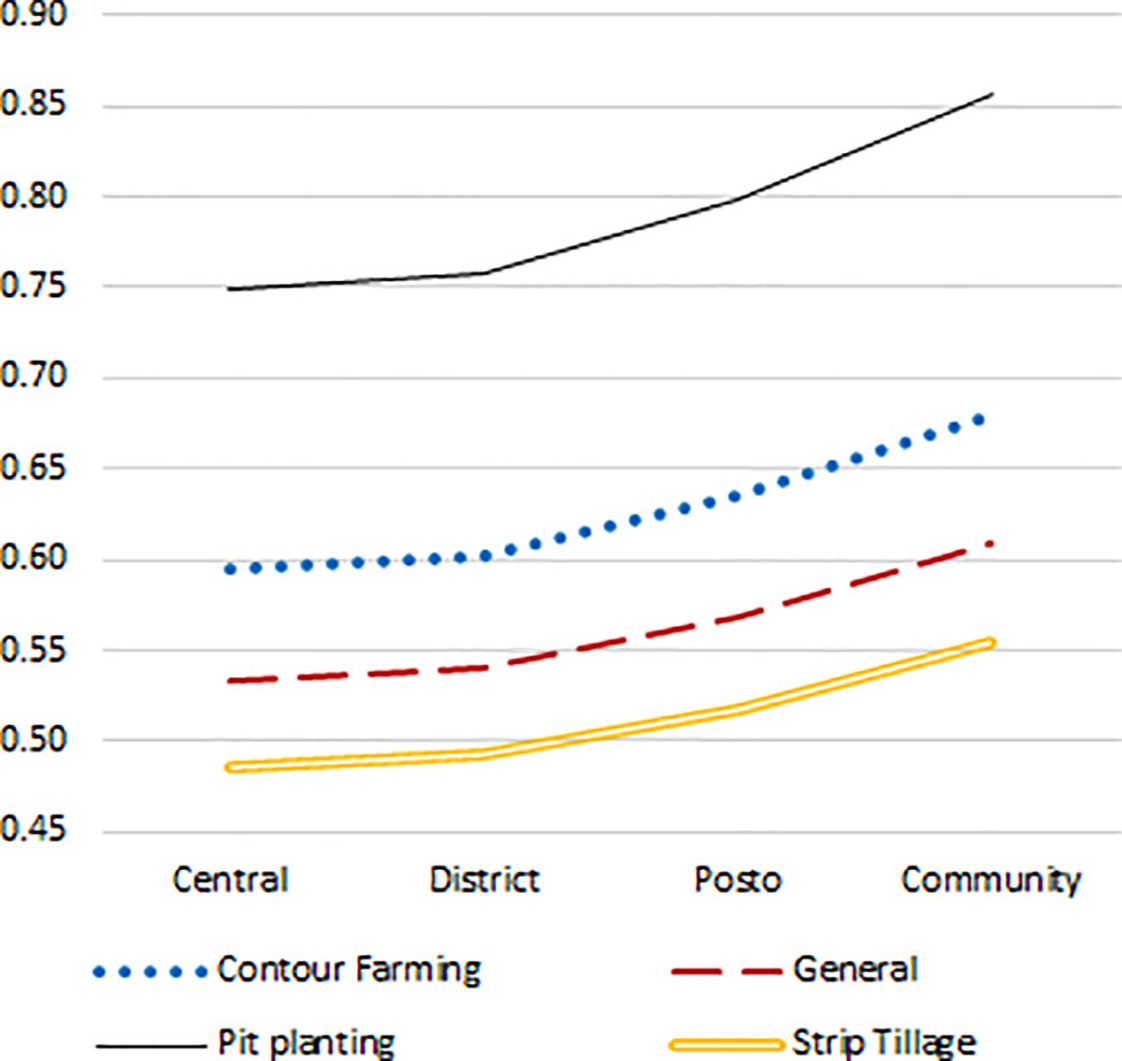
CGAAS: CERs of the gendered treatment as share of CERs of the general treatment. Notes: CGAAS = community-based gendered agricultural advisory services; CER = cost-effectiveness ratios. Underlying CERs pertain to cost-effectiveness of the programme in increasing knowledge about different sustainable land management (SLM) techniques: contour farming, pit planting, strip tillage, and general SLM knowledge. CERs are based on costs and impact by the time of the endline survey.

Fig 1 provides a visually efficient comparison of the gendered intervention’s CER with the overall intervention based on contact-farmers’ knowledge about (rather than awareness of) SLM practices. Each line reflects the ratio between the gendered intervention’s CER and the broad intervention’s CER, for the different SLM techniques and under different degrees of cost-inclusiveness. The *relative* cost-effectiveness of the gendered treatment vis-à-vis the overall intervention is always highest (that is, the ratio of the two CERs is lowest) for improving contact-farmers’ knowledge of strip tillage practices. The gendered treatment’s relative cost-effectiveness vis-à-vis the overall intervention is always lowest in regard to knowledge about the pit planting method. More importantly, the gendered treatment appears as having a higher cost-effectiveness than the overall intervention only for the narrowest method of accounting for costs.

### 3.6. Cost-effectiveness of agricultural advisory services in terms of farmers’ outcomes

The preceding section focused on the interventions’ cost-effectiveness for improving contact-farmers’ awareness of and skills with respect to agricultural conservation techniques. Given that the project’s objective was to improve the understanding and ultimately adoption of appropriate conservation practices among these farmers, who receive opportunities to learn from the contact-farmers, we also conduct the CEA with respect to them. Table 8 presents the results on the cost-effectiveness of the intervention components in improving ordinary (i.e. non-contact) farmers’ awareness in SLM. We primarily present results in terms of one SLM technique, pit planting, as the impact of advisory services had most impact on this farming approach (see for more detail the impact coefficients across multiple regressions in Section B in S1 Appendix). As heretofore, we present the CERs under different degrees of cost-inclusiveness. To obtain the count of key farmers in the communities where the intervention was carried out, information about the average household size for each locality was obtained both from Mozambique’s latest population census and from the aforementioned household sample survey. CERs based on both are presented, and the variation is only mild.

**Table 8:** CGAAS: Cost-effectiveness in increasing SLM farming methods awareness.

	Treatment arm	Central		District		Post		Community	
Gender		(1)	(2)	(1)	(2)	(1)	(2)	(1)	(2)
All	Basic tr.	5.40**	5.45**	4.21**	4.25**	1.93**	1.95**	1.01**	1.02**
	Gendered tr.	3.21***	3.22***	2.56***	2.57***	1.33***	1.33***	0.83***	0.83***
Men	Basic tr.	7.74**	7.80**	6.04**	6.09**	2.77**	2.79**	1.45**	1.46**
	Gendered tr.	5.83***	5.85***	4.66***	4.68***	2.42***	2.43***	1.51***	1.51***
Women	Basic tr.	15.26*	15.40*	11.91*	12.01*	5.46*	5.51*	2.86*	2.88*
	Gendered tr.	7.09***	7.11***	5.66***	5.68***	2.94***	2.95***	1.83***	1.84***

Notes: CGAAS = community-based gendered agricultural advisory services; SLM = sustainable land management. Values in 1,000 Mozambican meticais. Exchange rate prevailing in mid-2013: US$1 = 29.6 MZN. Cost-effectiveness ratios are based on costs and impact by the time of the midline survey. SLM farming technique pertains to pit planting. Columns 1 and 2 use coverage based on household size estimation from the latest available Mozambique population census and from the household survey, respectively. CERs are only reported for cases with statistically significant underlying effects (see further details in Section 2.6).

***, **, and * indicate statistical significance at the 1, 5, and 10 per cent levels, respectively.

As Table 8 shows, the gendered intervention—which trains and deploys a female, along with the male, contact-farmer in each treatment community—is more cost-effective than the intervention component that does not include the additional female contact-farmer. This holds irrespective of the cost-inclusiveness approach in CER calculation and is robust to the method of estimating the population of farmers subjected to the interventions. This is truly noteworthy and demonstrates the added value of CEA: in standard impact evaluation. While the experimental design limits our ability to interpret the benefits of the gendered treatment, in terms of the effect of having two trained contact farmers versus having an additional female contact farmer trained in the community, the CEA deems the gendered intervention more cost-effective in improving farmers’ information base about the SLM technique, in spite of its additional expenses.

What is true for farmers as a whole is also true when considering male and female farmers separately. The gendered treatment is more cost-effective in increasing women farmers’ awareness of the SLM technique and in increasing male farmers’ SLM awareness. The only exception to this conclusion derives from the cost-inclusion methodology that accounts exclusively for the most local costs: in that case, the CER for the ungendered treatment is slightly lower than that for the gendered treatment, but only for male farmers. This is partly due to the fact that a large part of the costs that derive from the addition of the second, female, contact-farmer accrues at this local level.

Finally, Table 9 gives the CERs analogous to Table 8, but for farmers’ adoption of, rather than information base about, the SLM technique. The impact evaluation coefficients were not consistently statistically significant for female farmers, and thus the table presents the CERs for all farmers and for male farmers. The ratios show findings fairly consistent with those in Table 8: at least for higher cost-inclusiveness, the gendered treatment is more cost-effective than the treatment arm including only male contact-farmers. However, the difference in cost-effectiveness is by far not as large as in the case of awareness of SLM practices. This is consistent with findings from the literature. While this study is, to the best of our knowledge, the first cost-effectiveness analysis on the topic concerned, we did identify other work with findings consistent with the difference we discern between gender differences in awareness, versus adoption, improved farming practices. For example, a study showed that in Niger, a clear gap prevailed between men’s and women’s use of rice farming technologies, but no significant gender gap was discerned in the level of knowledge about these technologies [22].

**Table 9:** CGAAS: Cost-effectiveness in increasing adoption of SLM farming technique.

	Treatment arm	Central		District		Post		Community	
Gender		(1)	(2)	(1)	(2)	(1)	(2)	(1)	(2)
All	Basic tr.	11.89*	11.99*	9.27*	9.35*	4.25*	4.29*	2.22*	2.24*
	Gendered tr.	9.52*	9.55*	7.61*	7.64*	3.95*	3.96*	2.47*	2.47*
Men	Basic tr.	13.51**	13.63**	10.54**	10.63**	4.84**	4.88**	2.53**	2.55**
	Gendered tr.	13.65**	13.69**	10.91**	10.95**	5.66**	5.68**	3.54**	3.55**

Notes: CGAAS = community-based gendered agricultural advisory services; SLM = sustainable land management. Values in 1,000 Mozambican meticais. Exchange rate prevailing in mid-2013: US$1 = 29.6 MZN. Cost-effectiveness ratios are based on costs and impact by the time of the midline survey. SLM farming technique pertains to pit planting. CERs are only reported for cases with statistically significant underlying effects (see further details in Section 2.6).

***, **, and * indicate statistical significance at the 1, 5, and 10 per cent levels, respectively.

Furthermore, the cost-effectiveness advantage of the gendered treatment is overturned for narrower definitions of costs (for example, costs inclusive through the post level and through the community level, for outcomes measured for all farmers.

### 3.7. How would cost-effectiveness change if the programme were scaled up?

In simulating the way that cost-effectiveness changes when a programme is scaled up, we need to be precise about the nature of the scale-up. As was explained earlier in Section 2.4, scaling up a given intervention component to the locality level means for our purposes that any given locality that originally contained one or more communities subjected to the intervention component is, after the scale-up, “saturated” by that intervention component. That is, scaling up implies that the treatment now takes place in each community of that locality. For the empirical implementation, we use additional information on the new jurisdictions which will receive the intervention after scale-up, especially demographic and population data from the population census, on the basis of which the costs of expanding into these areas are estimated (e.g. of $C_{ul}^{j}$ Eq 5; see Section 2.4 for more details). We assume that the marginal impact coefficients remain unvaried from the original intervention. Thus, the key drivers of evolution in cost-effectiveness derive from the extent to which some of the higher-tier costs are shared between the original programme areas and new expansion areas, as well as on the coverage that can be achieved in the new areas given the latters’ demographic and population characteristics.

Table 10 reflects a comparison of the CERs in Table 8 with their analogue after a scale-up to the locality level (upper panel) and after a scale-up to the administrative post level (lower panel). In particular, it shows the ratio of each CER resulting from a simulated scale-up of the intervention to the corresponding CER without scale-up in Table 8. For example, the ratio in the first row, column 1, shows that if the programme were scaled up within localities, the CER corresponding to the basic treatment would be only 60 per cent of the same CER before the scale-up, under the broadest degree of cost-inclusiveness (i.e. under inclusion of all costs up to the central level). In other words, after scaling up the programme it would become 40 per cent cheaper than in the original, pre-scale-up programme, to achieve the same effect. Any ratio above 1 in Table 10 suggests that achieving the same outcomes becomes more expensive after scale-up.

**Table 10:** CGAAS: CERs under scale-up scenarios as a share of CERs of original programme.

Gender	Treatment arm	Central	District	Post	Community
*Scale-up within localities*					
All	Basic tr.	0.600**	0.637**	0.832**	1.162**
	Gendered tr.	1.076***	1.144***	1.455***	1.844***
Men	Basic tr.	0.600**	0.636**	0.831**	1.160**
	Gendered tr.	1.079***	1.147***	1.459***	1.849***
Women	Basic tr.	0.601*	0.638*	0.833*	1.163*
	Gendered tr.	1.073***	1.140***	1.450***	1.838***
*Scale-up within posts/districts*					
All	Basic tr.	0.577**	0.616**	0.824**	1.175**
	Gendered tr.	0.905***	0.972***	1.283***	1.672***
Men	Basic tr.	0.577**	0.616**	0.824**	1.175**
	Gendered tr.	0.908***	0.975***	1.286***	1.676***
Women	Basic tr.	0.577*	0.615*	0.824*	1.175*
	Gendered tr.	0.903***	0.970***	1.280***	1.667***

Notes: CGAAS = community-based gendered agricultural advisory services; CER = cost-effectiveness ratios. All underlying ratios pertain to the cost-effectiveness of the programme in increasing awareness about the pit planting technique of sustainable land management. CERs are based on costs and impact by the time of the midline survey. CERs are only reported for cases with statistically significant underlying effects (see further details in Section 2.6).

***, **, and * indicate statistical significance at the 1, 5, and 10 per cent levels, respectively.

The results point to significant variation in the gains from scaling up, depending on which intervention type is being scaled up. Generally speaking, these gains are found to be greater when scaling up the basic treatment than they are in the case of the gendered treatment. For example, while the basic treatment’s CER after scale-up to the post level is only 58 per cent of the original (pre-scale-up) CER, the gendered treatment’s CER after such a scale-up is 91 per cent of its pre-scale-up analogue. This is in part driven by the fact that the population newly reached through expansion of the basic intervention is larger than the population newly reached through expansion of the gendered treatment. While the gains in cost-effectiveness in the process of scaling up vary importantly depending on what modality (gendered versus basic) of the programme is being scaled up, we do not see much contrast in gains in cost-effectiveness from scale-up when considering different target groups (male versus female farmers).

As seen in the above cases, gains can be had from scaling up in some cases. However, the results in Table 10 also make clear that scaling up need not improve cost-effectiveness. This is apparent, for example, in the evolution of the CER of the gendered treatment as it is scaled up to the locality level. Across all methods of cost-inclusiveness, the CER is larger after scale-up than before (i.e. all values in the upper panel are greater than 1 for the gendered modality). In these cases, aggregate benefits of the programme would expand to a lesser extent than aggregate costs in the course of the scale-up—this can be affected, for example, by a lower population density of beneficiaries in the newly reached communities relative to the density of the originally treated communities.

The results also show empirically that there may well be nonlinearities in cost-effectiveness in the process of scale-up. While as mentioned earlier there are gains from scaling the basic treatment to localities and further (small) gains to scaling it to posts, in other cases, cost-effectiveness could first decrease but then increase with further expansion. For example, scaling the gendered modality to localities results in a deterioration of cost-effectiveness (CER goes up by 7.6 per cent), but scaling it further to the post level instead improves cost-effectiveness of this modality (CER of gendered treatment scaled up to the post level is 90.5 per cent of original CER).

The degree of cost-inclusiveness in deriving CERs leads to strongly differing conclusions about the gains in cost-effectiveness of a programme as it is scaled up. The ratio of the scaled-up CER to the original CER becomes larger (in other words, the gains from scaling up are smaller, or the losses larger) the narrower the range of costs accounted for—that is, as one moves from column 1 to columns 2, 3, and 4. This follows from the fact that the narrower ways of accounting for costs leave out more cost components that contribute to the economies of scale from expansion.

The analysis above illustrates the extent of gains in cost-effectiveness of the agricultural extension programme were it to be expanded in scale. However, we may also want to ask whether the key conclusions drawn from the original programme’s CEA would obtain after the scale-up. Findings presented in Table 11 suggest not necessarily. The results here are the scaled-up analogue to results in Table 9, focusing on the cost-effectiveness of the intervention in terms of farmers’ adoption of SLM practices. In scenarios of programme expansion within localities, and expansion within posts, the basic treatment now emerges as more cost-effective than the gendered treatment. In the original programme (see Table 9), it was the reverse, at least for the main scenarios. This example, combined with the analysis presented in Table 10, shows that nonlinearities along the expansion path, as well as different degrees to which cost-effectiveness changes across modalities, can result after a scale-up in reversals of conclusions regarding which intervention modality is more cost-effective.

**Table 11:** CGAAS: Cost-effectiveness in increasing SLM practice adoption, under scale-up scenarios.

Gender	Treatment arm	Central	District	Post	Community
*Scale-up within localities*					
All	Basic tr.	7.14*	5.90*	3.54*	2.58*
	Gendered tr.	10.25*	8.71*	5.74*	4.55*
Men	Basic tr.	8.10**	6.70**	4.02**	2.93**
	Gendered tr.	14.74**	12.52**	8.26**	6.54**
*Scale-up within posts/districts*					
All	Basic tr.	6.85*	5.71*	3.51*	2.61*
	Gendered tr.	8.62*	7.40*	5.07*	4.12*
Men	Basic tr.	7.79**	6.49**	3.99**	2.97**
	Gendered tr.	12.39**	10.64**	7.28**	5.93**

Notes: CGAAS = community-based gendered agricultural advisory services. Values in 1,000 Mozambican meticais. Exchange rate prevailing in mid-2013: US$1 = 29.6 MZN. SLM farming technique pertains to pit planting. Cost-effectiveness ratios are based on costs and impact by the time of the midline survey. CERs are only reported for cases with statistically significant underlying effects (see further details in Section 2.6).

***, **, and * indicate statistical significance at the 1, 5, and 10 per cent levels, respectively.

### 3.8. Cost-effectiveness of the community-based land advisory services intervention

Table 12 presents the CERs for CGLAS outcomes that were determined statistically significant. As Table 12 shows, the cost per person of achieving outcomes related to knowledge and attitudinal changes with regard to land rights is moderate, ranging from approximately 14,000 to 43,000 Tanzanian shillings (TZS), or about $8.60 to $26.50, at the exchange rate of US$1 to 1,626 TZS that prevailed in mid-2014. At this point it needs underlining again that, similar to the agricultural advisory services programme, the very nature of the paralegal advisory services intervention does not lend itself to disaggregation of costs by the different outcomes that committing these costs bring about. Therefore, for example, the CER of 14.79 in Table 12 tells us that, accounting for the total costs of the intervention attributable to providing paralegal services to men, it takes about 14,790 TZS per man to improve his knowledge on land rights with regard to government expropriation—but the same total expenditures on providing services to men also brought about other additional outcomes for men, as seen in the table.

**Table 12:** CGLAS: Results of cost-effectiveness analysis, treatment based on assignment and compliance.

Outcomes	ITT estimation		TOT estimation	
	Men	Women	Men	Women
Believes wife should inherit		41.18**		42.99**
Aware of paralegal in village	20.49***	33.15***	21.63***	34.61***
Answered question correctly about who to approach in unresolved large land conflict	25.06**		26.46**	
Answered question correctly about government having the right to expropriate land for public use	14.79***	21.23**	15.52***	22.26**

Notes: CGLAS = community-based gendered land advisory services. Values in 1,000 Tanzanian shillings. Exchange rate prevailing in mid-2014: US$1 = 1,626 TZS. CERs are only reported for cases with statistically significant underlying effects (see further details in Section 2.6).

***, **, and * indicate statistical significance at the 1, 5, and 10 per cent levels, respectively.

Given the consistent methodology in deriving the CERs, these ratios can be compared against each other. A gender comparison of the ratios shows that in all cases of statistically significant results, the cost of bringing about any given outcome per man is lower than the cost per woman for the same outcome. For example, the cost per man of improving knowledge about the government expropriation of land is 69.7 per cent of the cost of educating a woman on this topic (derived from the cost-effectiveness ratios in the last row of Table 12, i.e. 14.79/21.23, or 15.52/22.26). Similarly, the cost to raise awareness among men about the presence of paralegals in the village (about 20,500 TZS per man) is approximately 61.8 per cent of the cost to raise such awareness among women (about 33,200 TZS per woman).

There are a number of factors that can contribute to the lower delivery cost per outcome for men than for women. It is for example well known that social networks are an important vehicle for information diffusion, especially in rural low-income settings where other means of information dissemination are sparse. This also includes the type of information considered concerning agricultural practices [23] and land tenure rights [24]. In these settings, advice on sustainable land management and land rights issues, even when offered through community-based extension workers that are attentive to reaching both men and women farmers, may travel differentially through gendered social networks, resulting in further diffusion that may vary between men and women. This has, for example, been documented a number of empirical cases across developing countries [25, 26].

We do however find that the gender gap in cost-effectiveness is quantitatively somewhat narrower than if one considered the gender gap merely in terms of the marginal effects (that is, without accounting for delivery costs). For example, the marginal effect of the intervention on women’s knowledge about government expropriation is 63 per cent of the corresponding marginal effect on men (derived as the ratio between the corresponding impact coefficients, not shown in the CER tables), which reflects a larger gender gap than the aforementioned 69.7 per cent. The difference in the gender gap of the CERs versus just of the marginal effect is because the CERs take into account not only total impact, which is affected by differential population size between men and women in the treatment areas, but also total costs—affected among other things by differential effort placed in providing land paralegal services to men versus women, as discussed above in the context of the cost and noncost data used and analysed.

Finally, Table 12 demonstrates that the CERs are fairly robust to differences in the underlying regression analysis—that is, whether it is based on TOT or ITT effects. The TOT-based cost-effectiveness ratios are slightly larger. Although the underlying TOT impact coefficients are, as is to be expected, larger than those of the ITT regressions, this is slightly more than offset by cost features that render service provision somewhat more expensive in the 16% of communities that no longer had paralegals by the time of the endline. This results in TOT CERs that are higher, however only by about 4% to 6%.

Table 13 presents the CERs that account for spillover of the project beyond the intervention areas. First, the fact that in the first two columns there exist some statistically significant effects based on which CERs could be derived suggests that there are spillover effects of the paralegal aid programme. Those need to be accounted for, and we do so in the second two columns, which report the CERs based on programme impacts comparing the treatment area to “pure control” areas—that is, after having removed the villages in high proximity to the intervention villages.

**Table 13:** CGLAS: Results of cost-effectiveness analysis accounting for spillovers.

Outcomes	Spillover effects		Pure effects (control excludes spillover areas)	
	Men	Women	Men	Women
Believes wife should inherit				34.50**
Believes land and housing tribunal treats cases fairly	107.77**	112.73**	32.26*	36.04**
Aware of paralegal in village			17.83***	38.30***
Answered question correctly about who to approach in unresolved large land conflict			30.24**	
Answered question correctly about recognised son’s entitlement to inheritance	79.34**			
Answered question correctly about government having the right to expropriate land for public use			14.27**	19.16**
Attended seminar on legal rights in the last 12 months		487.33**		

Notes: CGLAS = community-based gendered land advisory services. Values in 1,000 Tanzanian shillings. Exchange rate prevailing in mid-2014: US$1 = 1,626 TZS. CERs are only reported for cases with statistically significant underlying effects (see further details in Section 2.6).

***, **, and * indicate statistical significance at the 1, 5, and 10 per cent levels, respectively.

The CERs in the first two columns of Table 13 are naturally much larger than those in the second two columns, since the former reflect the cost of the total intervention but consider its improvement of knowledge, attitudes, and practices per person only in the areas outside of, but proximate to, the treatment areas. With such spillover areas being relatively small in scale and the costs incurred by the intervention pertaining to a much larger treatment area, the significantly larger CERs are expected.

A gender comparison of the CERs with regard to spillover areas wherever such a comparison is possible—namely, on the cost-effectiveness of increasing the sense of fair treatment by the land tribunal among men and women in spillover villages—shows that here the gender gap in cost-effectiveness dramatically narrows. The cost of increasing the experience of fair treatment by the land tribunal among men is nearly equal to—specifically, 95.6 per cent that of—the cost of doing so among women in spillover villages. The fact that the cost-effectiveness gender gap is much larger when considering the direct effects of the programme than the cost-effectiveness gender gap of the indirect effects of the programme (that is, in spillover areas) is suggestive of the fact that indirect diffusion of the programme’s benefits may have more gender-equal “bang for the buck.” It is, however, important to consider this possibility with caution, given that spillover effects are identified only on a few outcomes, and only in one case can the cost-effectiveness gender gap be measured. Finally, as was true in regard to the CERs in Table 12, across all gender comparisons in the CERs that account for potential for spillover in Table 13, the gender gap is always narrower than the equivalent gender comparisons of just the marginal effects.

Finally, since some assumptions were necessary in the CEA—in particular, concerning the use of a discount rate and the useful life of capital costs—it is important to examine how widely the CERs vary with changes in these assumptions. As mentioned earlier in the subsection on periodicity, the main results in Tables 12 and 13 are based on an annual discount rate of 3 per cent and an assumed useful life of capital items double that in formal estimates for capital items in the United States, and 10 years for the Mozambique analysis. Tables Aa–Ad in S1 Appendix consider results for large variations in these assumptions for some of the main results: discount rates of 1 per cent and 10 per cent, useful lives of capital equipment in Tanzania that are 1 times and 4 times those in the United States, and useful lives half and double the estimated useful life of 10 years in Mozambique. It is apparent that our CERs are quite robust to such large changes across the four scenarios of discount rate and capital durability assumptions.

## 4. Discussion

For research to inform policy and programmatic work in development, it is necessary to go beyond examining the impact of programmes to measuring their cost-effectiveness. While this message has reached scholars investigating health and education issues, it rarely is addressed in studies focusing on the agricultural sector. We address that gap, specifically in the context of two interventions that use community-based trainers to increase female and male farmers’ awareness, knowledge, and practices with regard to agricultural production and land rights. This study lays out in detail the methodological considerations in CEA in these contexts and presents results for the two programmes. In so doing, we also take first steps to address another concern of major import for policy makers, namely, how cost-effectiveness may change as programmes such as these are scaled up.

The agricultural advisory services programme in Mozambique employed two modalities in conveying messages on sustainable land management practices to smallholders, with those two modalities differing in the gender sensitivity in selection of contact-farmers to provide advice to their fellow farmers in their respective communities. In contrast, the land advisory services programme consisted of a single modality. The nature of the agricultural extension programme thus enabled a comparison in cost-effectiveness across two modalities of that intervention. Our analysis shows that the gender-sensitive modality is more cost-effective than the basic modality, and that it is also more cost-effective than the overall intervention (that is, when not distinguishing between the two modalities). Comparing the two modalities in terms of their cost-effectiveness, and not only their impact, is particularly useful in light of the fact that the addition of a second (female) contact-farmer in the gendered modality also brings with it higher costs than the basic treatment that has only one (male) contact-farmer per community.

Including a female contact-farmer to work side by side with the male contact-farmer more cost-effectively increases the awareness as well as knowledge of farmers about various SLM practices, such as contour farming, pit planting, and row planting. The stronger cost-effectiveness of the gendered modality holds up when considering awareness, knowledge, and adoption of farmers overall—not merely female farmers as recipients of advice. This advantage of the gender-sensitive modality in community-based agricultural advisory services is present quite consistently across different assumptions and variations in costing methodology. Exceptions where the basic treatment is more cost-effective are few—for example, under the narrowest inclusion of costs, namely, only those accruing at the community level, and when considering awareness and knowledge of male farmers. Also, the basic modality was more cost-effective than the gendered modality in bringing about SLM adoption by male farmers.

While gender sensitivity in the supply of community-based agricultural advisory services generally has been shown to pay off, our findings indicate that, for *any given* modality, increasing male farmers’ awareness, knowledge, and adoption of SLM practices is more cost-effective than strengthening SLM practices among women farmers. This conclusion is robust to the method used in terms of cost-inclusiveness across tiers. The analogous is the case in the community-based land rights advisory services project. In that project, too, efforts to improve knowledge and awareness of land rights among men are more cost-effective than increasing women’s knowledge about land rights. The cost-effectiveness gender-gap is however narrower for awareness than adoption of improved SLM practices.

The fact that it is cheaper to affect the outcomes of male versus female community members may be an outgrowth of gender differentials in the depth of knowledge and literacy at the outset. For example, considering men and women who at baseline cannot correctly answer which agencies deal with land conflict, the men’s knowledge about this issue may be closer to the threshold of being correct than women’s knowledge, and thus more time and effort, and thus costs, may be required to ensure women have the correct understanding about land-related agencies than to achieve this with respect to men. Furthermore, there are of course societal benefits to gender equity that are not explicitly captured in the framework of this paper. The narrowed gender-gap in improving awareness of improved farming practices, versus adoption of such practices, is consistent with findings elsewhere. An extensive review of the literature on the gendered nature of agricultural technology adoption identifies as primary causes for women’s slower adoption lower levels of access to complementary inputs and services, as well as, further upstream, women’s lower levels of participation in priority-setting and decisionmaking at the community level regarding which agricultural technologies and innovations should receive extension and other support [27].

Given the importance to programmatic work of how a project’s cost-effectiveness may vary if it is scaled, we simulate the changes in CERs under various scenarios of scaling up the agricultural advisory services programme (scale-up analysis was not undertaken for CGLAS, given limited granularity of cost data across tiers). Expansion of the basic modality of the programme leads to improvements in cost-effectiveness, while the gendered modality displays nonlinearities along the expansion path: cost-effectiveness declines with initial expansion, then increases with further-reaching scale-up. Comparison of the cost-effectiveness across the two modalities after expansion shows that the earlier comparison becomes reversed: following scale-up, the gendered treatment performs worse in terms of cost-effectiveness than the basic treatment. These simulated results on how cost-effectiveness changes with programme expansion—the positive findings as well as the less encouraging ones from the perspective of the contribution of the gender-sensitive modality in service delivery—can both serve as first indications that a naïve assumption of linear and proportional application of the original levels of cost-effectiveness to a scaled-up programme may serve programmatic work poorly.

With academic studies conducting cost-effectiveness analyses of agricultural interventions in development being rather sparse, this study arguably remains with some constraints and limitations, which however point to areas for future research. First, as with any CEA or even just impact evaluation of an intervention that achieves multiple outcomes, there are challenges in separately identifying complementary effects across the outcomes—especially when specific intervention modalities cannot be isolated or assumed to achieve entirely separate outcomes. There are well established methods in experimental evaluation to derive cross-effects across modalities, and to assess interaction effects of various characteristics of the beneficiaries, etc. However, a rigorous analysis that isolates and quantifies the synergies of achieving multiple *outcomes* through the *same* intervention faces significant analytical and data challenges. In our study, it may have been of interest to allow for CERs for a given outcome to be influenced by the achievement of another outcome. In addition to the limitations of doing so in the CER denominator (i.e. in the impact evaluation, the reason why this cannot be done in the numerator, or in the assessment of costs) was explained in various places in this paper, including at the beginning of Section 3.5. Nonetheless, future work should seek to derive methodological approaches that may at least go some way in measuring the size of synergies across outcomes.

For analysis of cost-effectiveness under scenarios of scaled-up projects, more work is needed to deepen the simulation, with several variations in the assumptions, and to conduct it in other contexts in a search for more widely generalisable patterns. Given that the intervention did not in fact take place in the simulated scale-up areas, we do not have intervention costs and intervention impact coefficients that can be directly derived and applied. However, additional alternative methods than those employed in this study can be explored to estimate what the costs and impacts would be under an imagined expansion.

## Supporting information

S1 Appendix A. Cost-effectiveness and cost-benefit analysis
Table A. Comparison between cost-benefit analysis (CBA) and cost-effectiveness analysis
*Source*: *Authors’ analysis*.
B. Impact evaluation coefficients
Table B. Effect of SLM training intervention (basic and gendered treatments) on contact farmers.
*Source*: *Mozambique Household Survey and Contact Farmer Survey*, *2012*, *2013*. Notes: SLM = sustainable land management. Regressions include the following variables: a constant, age, completed at least primary school dummy, single dummy, number of children, total landholdings, the number of rooms in the household, the number of hours worked by the CF at baseline, district indicators, and incentive treatment indicators. Robust standard errors in parentheses. ***, **, and * indicate significance at the 1, 5, and 10 percent critical level.
Table C. Effect of SLM training intervention (any treatment) on contact farmers.
*See source and notes of Table B*.
Table D. Effect of SLM training intervention (basic and gendered treatments) on contact farmers' SLM awareness.
*See source and notes of Table B*.
Table E. Effect of SLM training intervention (any treatment) on contact farmers' SLM awareness.
*See source and notes of Table B*.
Table F. Effect of SLM training intervention (basic and gendered treatments) on contact farmers' SLM knowledge.
*See source and notes of Table B*.
Table G. Effect of SLM training intervention (any treatment) on contact farmers' SLM knowledge.
*See source and notes of Table B*.
Table H. Effect of SLM training intervention (basic and gendered treatments) on contact farmers' SLM adoption.
*See source and notes of Table B*.
Table I. Effect of SLM training intervention (any treatment) on contact farmers' SLM adoption.
*See source and notes of Table B*.
Table J. Effect of SLM training intervention (basic and gendered treatments) on farmers.
*Source*: *Mozambique Household Survey*, *2012*, *2013*. Notes: SLM = sustainable land management. Regressions include the following variables: a constant, age, completed at least primary school dummy, single dummy, widow dummy, number of children, total landholdings, the number of rooms in the household, the number of hours worked by the CF at baseline, district indicators, and incentive treatment indicators. Standard errors clustered at the community level in parentheses. ***, **, and * indicate significance at the 1, 5, and 10 percent critical level.
Table K. Effect of SLM training intervention (any treatment) on farmers.
*See source and notes of Table J*.
Table L. Effect of SLM training intervention (any treatment) on farmers' SLM awareness.
*See source and notes of Table J*. The corresponding results on the separate (i.e. gendered and basic) treatment effects are captured in [28].
Table M. Effect of SLM training intervention (any treatment) on farmers' SLM knowledge.
*See source and notes of Table J*. The corresponding results on the separate (i.e. gendered and basic) treatment effects are captured in [28].
Table N. Effect of SLM training intervention (any treatment) on farmers' SLM adoption.
*See source and notes of Table J*. The corresponding results on the separate (i.e. gendered and basic) treatment effects are captured in [28].
Table O. Effect of SLM training intervention (basic and gendered treatments) on pooled farmers.
*Source*: *Mozambique Household Survey*, *2012*, *2013*. Notes: SLM = sustainable land management. Regressions include the following variables: a constant, male dummy, age, completed at least primary school dummy, single dummy, widow dummy, number of children, total landholdings, the number of rooms in the household, the number of hours worked by the CF at baseline, district indicators, and incentive treatment indicators. Standard errors clustered at the community level in parentheses. ***, **, and * indicate significance at the 1, 5, and 10 percent critical level.
Table P. Effect of SLM training intervention (any treatment) on pooled farmers.
*See source and notes of Table O*.
Table Q. Effect of SLM training intervention (basic and gendered treatments) on pooled farmers' SLM awareness.
*See source and notes of Table O*.
Table R. Effect of SLM training intervention (any treatment) on pooled farmers' SLM awareness.
*See source and notes of Table O*.
Table S. Effect of SLM training intervention (basic and gendered treatments) on pooled farmers' SLM knowledge.
*See source and notes of Table O*.
Table T. Effect of SLM training intervention (any treatment) on pooled farmers' SLM knowledge.
*See source and notes of Table O*.
Table U. Effect of SLM training intervention (basic and gendered treatments) on pooled farmers' SLM adoption.
*See source and notes of Table O*.
Table V. Effect of SLM training intervention (any treatment) on pooled farmers' SLM adoption.
*See source and notes of Table O*.
Table W. Results of cost-effectiveness analysis of land advisory services, treatment based on assignment and compliance.
*Source: Tanzania Household Survey and Community Survey, 2013, 2014. [29].* Notes: ITT = intent to treat; TOT = treatment on the treated. Standard errors in parentheses. Baseline marital status, education, age, age-squared, land tercile categories, and district variables included as control variables.***, **, and * indicate significance at the 1, 5, and 10 percent critical level.
Table X. Results of cost-effectiveness analysis of land advisory services, accounting for spillovers.
*Source: Tanzania Household Survey and Community Survey, 2013, 2014. [29].* Notes: Standard errors in parentheses. Baseline means and standard errors computed using inverse probability weights. Baseline marital status, education, age, age-squared, land tercile categories, and district variables included als control variables. ***, **, and * indicate significance at the 1, 5, and 10 percent critical level.
C. Additional descriptive statistics on costs and coverage
Table Y. Summary of costs of the agricultural SLM advisory services programme, by year and tier.
Note: Values in 1,000 Mozambican meticais. Exchange rate prevailing in mid-2013: US$1 = 29.6 MZN.
Table Z. Coverage under scale-up scenarios of the of agricultural SLM advisory services programme.
Notes: Given that in our project area each treatment arm is present in at least one community of each post, three features hold: for each district (1) the number of posts involving the basic treatment and the gendered treatment is identical to the total number of posts under both scale-up scenarios; (2) in the scale-up to posts/districts scenario, the number of communities and farmers under the basic treatment is equal to the number under gendered treatment; and (3) in the scale-up to posts/districts scenario, the number of localities involving a single treatment arm is identical to the total number of localities.
D. Sensitivity analysis on discount rate and capital durability
Table Aa. Cost-effectiveness of the gendered treatment of the SLM programme in increasing contact farmers’ SLM knowledge. Note: SLM = sustainable land management. Values in 1,000 Mozambican meticais. Exchange rate prevailing in mid-2013: US$1 = 29.6 MZN. Cost-effectiveness ratios are based on costs and impact by the time of the endline survey. Full cost-inclusiveness (corresponding to the “central” column in Table 6). Sensitivity analysis considers halving (to 5) and doubling (to 20) the assumed useful life of 10 years of capital items in the Mozambique programme. For these, sensitivity is also reported to variations in the assumed discount rate of 3%, namely one third that (1%) and more than three times that (10%). CERs are only reported for cases with statistically significant underlying effects (see further details in Section 2.6). ***, **, and * indicate statistical significance at the 1, 5, and 10 per cent levels, respectively.
Table Ab. Cost-effectiveness in increasing awareness of the pit planting technique of SLM.
Note: SLM = sustainable land management. Values in 1,000 Mozambican meticais. Exchange rate prevailing in mid-2013: US$1 = 29.6 MZN. Cost-effectiveness ratios are based on costs and impact by the time of the midline survey. Coverage based on household size estimation from the household survey. Full cost-inclusiveness (corresponding to the “central” column in Table 8). Sensitivity analysis considers halving (to 5) and doubling (to 20) the assumed useful life of 10 years of capital items in the Mozambique programme. For these, sensitivity is also reported to variations in the assumed discount rate of 3%, namely one third that (1%) and more than three times that (10%). CERs are only reported for cases with statistically significant underlying effects (see further details in Section 2.6). ***, **, and * indicate statistical significance at the 1, 5, and 10 per cent levels, respectively.
Table Ac. Cost-effectiveness in increasing adoption of pit planting of SLM, under scale-up scenarios.
Note: Values in 1,000 Mozambican meticais. Exchange rate prevailing in mid-2013: US$1 = 29.6 MZN. Cost-effectiveness ratios are based on costs and impact by the time of the midline survey. Full cost-inclusiveness (corresponding to the “central” column in Table 11). Sensitivity analysis considers halving (to 5) and doubling (to 20) the assumed useful life of 10 years of capital items in the Mozambique programme. For these, sensitivity is also reported to variations in the assumed discount rate of 3%, namely one third that (1%) and more than three times that (10%). CERs are only reported for cases with statistically significant underlying effects (see further details in Section 2.6). ***, **, and * indicate statistical significance at the 1, 5, and 10 per cent levels, respectively.
Table Ad. Sensitivity analysis on discount rate and capital durability in paralegal land advisory services programme.
Note: Cost-effectiveness ratio values in 1,000 Tanzanian shillings. Exchange rate prevailing in mid-2014: US$1 = 1,626 TZS. The figures in the column “Useful capital life” signify the multipliers used on US government indicators of the length of life of various capital equipment and items. Thus, in rows with the number 1, the US indicators are used in the Tanzania data. In rows with the number 4, the length of equipment life in the data is assumed to be four times that in US accounting standards. CERs are only reported for cases with statistically significant underlying effects (see further details in Section 2.6). ***, **, and * indicate statistical significance at the 1, 5, and 10 per cent levels, respectively.
(DOCX)
