# Supplementary material for: Cost-effectiveness of community-based gendered advisory services to farmers: Analysis in Mozambique and Tanzania
Source: PLoS One. 2019 Mar 20;14(3):e0211448. doi: 10.1371/journal.pone.0211448 (PMC6426202; doi:10.1371/journal.pone.0211448)
Supplement: S1 Appendix — A. Cost-effectiveness and cost-benefit analysis Table A. Comparison between cost-benefit analysis (CBA) and cost-effectiveness analysis Source: Authors’ analysis. B. Impact evaluation coefficients Table B. Effect of SLM training intervention (basic and gendered treatments) on contact farmers. Source: Mozambique Household Survey and Contact Farmer Survey, 2012, 2013. Notes: SLM = sustainable land management. Regressions include the following variables: a constant, age, completed at least primary school dummy, single dummy, number of children, total landholdings, the number of rooms in the household, the number of hours worked by the CF at baseline, district indicators, and incentive treatment indicators. Robust standard errors in parentheses. ***, **, and * indicate significance at the 1, 5, and 10 percent critical level. Table C. Effect of SLM training intervention (any treatment) on contact farmers. See source and notes of Table B. Table D. Effect of SLM training intervention (basic and gendered treatments) on contact farmers' SLM awareness. See source and notes of Table B. Table E. Effect of SLM training intervention (any treatment) on contact farmers' SLM awareness. See source and notes of Table B. Table F. Effect of SLM training intervention (basic and gendered treatments) on contact farmers' SLM knowledge. See source and notes of Table B. Table G. Effect of SLM training intervention (any treatment) on contact farmers' SLM knowledge. See source and notes of Table B. Table H. Effect of SLM training intervention (basic and gendered treatments) on contact farmers' SLM adoption. See source and notes of Table B. Table I. Effect of SLM training intervention (any treatment) on contact farmers' SLM adoption. See source and notes of Table B. Table J. Effect of SLM training intervention (basic and gendered treatments) on farmers. Source: Mozambique Household Survey, 2012, 2013. Notes: SLM = sustainable land management. Regressions include the following variables: a consta [file pone.0211448.s001.docx]

# SUPPORTING INFORMATION

# S1 APPENDIX

**A. Cost-effectiveness and cost-benefit analysis**

| Table A. Comparison between cost-benefit analysis (CBA) and cost-effectiveness analysis | | |
| --- | --- | --- |
| Features | CBA | CEA |
| Main objective as an economic evaluation technique | Mainly compares the cost of a programme/project/  Intervention to the benefit achieved | Mainly compares the cost per outcome of two or more programmes/ projects/ interventions |
| Costs are measured in: | Monetary terms | Monetary terms |
| Outcomes are measured in: | Monetary terms | Non-monetary terms |
| Range of outcomes usually considered for the technique: | Limited, namely to those that can be properly expressed in monetary terms | Wider range of outcomes can be considered, as outcomes need not be assigned an economic value |
| Accounting for externalities and complementarities | Easer to account for | Harder to account for |
| Types of comparisons that can be made in the analysis | Diverse programmes/ projects can be compared to one-another | Programmes/ projects compared to one another based in terms of the same outcomes achieved |
| *Source: Authors’ analysis.* | | |

**B. Impact evaluation coefficients**

| Table B. Effect of SLM training intervention (basic and gendered treatments) on contact farmers. | | | | | | | | | | | | |
| --- | --- | --- | --- | --- | --- | --- | --- | --- | --- | --- | --- | --- |
|  | Midline | | | | |  | | Endline | | | | |
|  | Ctrl. | Basic treatment | Gendered treatment | N | Adj. |  | Ctrl. | | Basic treatment | Gendered treatment | N | Adj. |
|  | Mean |  |  |  | R2 |  | Mean | |  |  |  | R2 |
| # of techniques known by name | 5.214 | 0.132 | 0.423 | 179 | 0.005 |  | 5.024 | | 0.800 | 1.593* | 168 | 0.156 |
|  |  | (0.521) | (0.628) |  |  |  |  | | (0.627) | (0.706) |  |  |
| Knowledge score | 0.642 | -0.028 | 0.006 | 179 | -0.022 |  | 0.661 | | 0.044 | 0.111** | 168 | 0.164 |
|  |  | (0.069) | (0.068) |  |  |  |  | | (0.028) | (0.026) |  |  |
| # of techniques adopted on any plot | 5.429 | 0.666 | 0.998* | 179 | 0.017 |  | 4.000 | | 0.384* | 0.936** | 168 | 0.088 |
|  |  | (0.331) | (0.447) |  |  |  |  | | (0.180) | (0.205) |  |  |
| *Source: Mozambique Household Survey and Contact Farmer Survey, 2012, 2013.* Notes: SLM = sustainable land management. Regressions include the following variables: a constant, age, completed at least primary school dummy, single dummy, number of children, total landholdings, the number of rooms in the household, the number of hours worked by the CF at baseline, district indicators, and incentive treatment indicators. Robust standard errors in parentheses. ***, **, and * indicate significance at the 1, 5, and 10 percent critical level. | | | | | | | | | | | | |

| Table C. Effect of SLM training intervention (any treatment) on contact farmers. | | | | | | | | | |
| --- | --- | --- | --- | --- | --- | --- | --- | --- | --- |
|  | Midline | | | |  | Endline | | | |
|  | Ctrl. | Any treatment | N | Adj. |  | Ctrl. | Any treatment | N | Adj. |
|  | Mean |  |  | R2 |  | Mean |  |  | R2 |
| # of techniques known by name | 5.214 | 0.280 | 179 | 0.005 |  | 5.024 | 1.176 | 168 | 0.122 |
|  |  | (0.560) |  |  |  |  | (0.619) |  |  |
| Knowledge score | 0.642 | -0.011 | 179 | -0.025 |  | 0.661 | 0.076** | 168 | 0.095 |
|  |  | (0.068) |  |  |  |  | (0.026) |  |  |
| # of techniques adopted | 5.429 | 0.834* | 179 | 0.016 |  | 4.000 | 0.646** | 168 | 0.072 |
|  |  | (0.359) |  |  |  |  | (0.168) |  |  |
| *See source and notes of S2a Table.* | | | | | | | | | |

| Table D. Effect of SLM training intervention (basic and gendered treatments) on contact farmers' SLM awareness. | | | | | | | | | | | |
| --- | --- | --- | --- | --- | --- | --- | --- | --- | --- | --- | --- |
|  | Midline | | | | |  | Endline | | | | |
|  | Ctrl. | Basic treatment | Gendered treatment | N | Adj. |  | Ctrl. | Basic treatment | Gendered treatment | N | Adj. |
|  | Mean |  |  |  | R2 |  | Mean |  |  |  | R2 |
| Mulching | 0.952 | 0.016 | 0.056 | 179 | 0.053 |  | 0.952 | -0.017 | 0.022 | 168 | 0.075 |
|  |  | (0.037) | (0.033) |  |  |  |  | (0.055) | (0.068) |  |  |
| Strip tillage | 0.548 | -0.007 | -0.081 | 179 | 0.073 |  | 0.595 | -0.008 | 0.224 | 168 | 0.097 |
|  |  | (0.125) | (0.146) |  |  |  |  | (0.106) | (0.111) |  |  |
| Pit planting | 0.714 | -0.011 | 0.179 | 179 | 0.095 |  | 0.667 | 0.203 | 0.255** | 168 | 0.029 |
|  |  | (0.107) | (0.156) |  |  |  |  | (0.105) | (0.075) |  |  |
| Contour farming | 0.548 | 0.024 | 0.060 | 179 | 0.014 |  | 0.381 | 0.244* | 0.319** | 168 | 0.040 |
|  |  | (0.163) | (0.151) |  |  |  |  | (0.104) | (0.096) |  |  |
| Crop rotation | 0.857 | 0.026 | 0.015 | 179 | -0.032 |  | 0.738 | 0.054 | 0.080 | 168 | 0.007 |
|  |  | (0.106) | (0.129) |  |  |  |  | (0.129) | (0.153) |  |  |
| Row planting | 0.286 | 0.062 | 0.076 | 179 | 0.038 |  | 0.357 | 0.183* | 0.366** | 168 | 0.084 |
|  |  | (0.083) | (0.118) |  |  |  |  | (0.081) | (0.081) |  |  |
| *See source and notes of S2a Table.* | | | | | | | | | | | |

| Table E. Effect of SLM training intervention (any treatment) on contact farmers' SLM awareness. | | | | | | | | | | |
| --- | --- | --- | --- | --- | --- | --- | --- | --- | --- | --- |
|  | Midline | | | |  | Endline | | | | |
|  | Ctrl. | Any treatment | N | Adj. |  | Ctrl. | Any treatment | N | Adj. |  |
|  | Mean |  |  | R2 |  | Mean |  |  | R2 |  |
| Mulching | 0.952 | 0.037 | 179 | 0.050 |  | 0.952 | 0.002 | 168 | 0.073 |  |
|  |  | (0.033) |  |  |  |  | (0.061) |  |  |  |
| Strip tillage | 0.548 | -0.044 | 179 | 0.074 |  | 0.595 | 0.102 | 168 | 0.057 |  |
|  |  | (0.136) |  |  |  |  | (0.098) |  |  |  |
| Pit planting | 0.714 | 0.086 | 179 | 0.060 |  | 0.667 | 0.228* | 168 | 0.032 |  |
|  |  | (0.130) |  |  |  |  | (0.086) |  |  |  |
| Contour farming | 0.548 | 0.043 | 179 | 0.019 |  | 0.381 | 0.280** | 168 | 0.041 |  |
|  |  | (0.155) |  |  |  |  | (0.090) |  |  |  |
| Crop rotation | 0.857 | 0.020 | 179 | -0.026 |  | 0.738 | 0.066 | 168 | 0.013 |  |
|  |  | (0.116) |  |  |  |  | (0.134) |  |  |  |
| Row planting | 0.286 | 0.069 | 179 | 0.043 |  | 0.357 | 0.270*** | 168 | 0.062 |  |
|  |  | (0.099) |  |  |  |  | (0.056) |  |  |  |
| *See source and notes of S2a Table.* | | | | | | | | | |  |

| Table F. Effect of SLM training intervention (basic and gendered treatments) on contact farmers' SLM knowledge. | | | | | | | | | | | | | | |
| --- | --- | --- | --- | --- | --- | --- | --- | --- | --- | --- | --- | --- | --- | --- |
|  | Midline | | | | | | |  | | Endline | | | | |
|  | Ctrl. | Basic treatment | Gendered treatment | N | Adj. | Ctrl. |  | | Basic treatment | | Gendered treatment | N | Adj. |  |
|  | Mean |  |  |  | R2 | Mean |  | |  |  |  |  | R2 |  |
| Mulching | 0.833 | 0.049 | 0.045 | 179 | 0.022 | 0.952 |  | | 0.028 | | 0.038* | 168 | -0.002 |  |
|  |  | (0.047) | (0.029) |  |  |  |  | | (0.020) | | (0.015) |  |  |  |
| Strip tillage | 0.460 | -0.001 | 0.063 | 179 | -0.019 | 0.563 |  | | 0.059 | | 0.210*** | 168 | 0.081 |  |
|  |  | (0.105) | (0.122) |  |  |  |  | | (0.058) | | (0.039) |  |  |  |
| Pit planting | 0.798 | -0.118 | 0.049 | 179 | 0.064 | 0.798 |  | | 0.122* | | 0.131* | 168 | 0.063 |  |
|  |  | (0.124) | (0.156) |  |  |  |  | | (0.052) | | (0.059) |  |  |  |
| Contour farming | 0.524 | 0.025 | 0.077 | 179 | -0.007 | 0.516 |  | | 0.149 | | 0.271** | 168 | 0.096 |  |
|  |  | (0.167) | (0.136) |  |  |  |  | | (0.103) | | (0.094) |  |  |  |
| Crop rotation | 0.540 | -0.060 | -0.085 | 179 | -0.021 | 0.595 |  | | 0.069 | | 0.126* | 168 | 0.047 |  |
|  |  | (0.063) | (0.059) |  |  |  |  | | (0.050) | | (0.053) |  |  |  |
| Row planting | 0.476 | -0.129 | -0.126 | 179 | 0.032 | 0.143 |  | | 0.036 | | 0.129 | 168 | 0.001 |  |
|  |  | (0.147) | (0.102) |  |  |  |  | | (0.126) | | (0.119) |  |  |  |
| *See source and notes of S2a Table.* | | | | | | | | | | | | | |  |

| Table G. Effect of SLM training intervention (any treatment) on contact farmers' SLM knowledge. | | | | | | | | | | |
| --- | --- | --- | --- | --- | --- | --- | --- | --- | --- | --- |
|  | Midline | | | |  | Endline | | | | |
|  | Ctrl. | Any treatment | N | Adj. |  | Ctrl. | Any treatment | N | Adj. |  |
|  | Mean |  |  | R2 |  | Mean |  |  | R2 |  |
| Mulching | 0.833 | 0.047 | 179 | 0.027 |  | 0.952 | 0.033 | 168 | 0.002 |  |
|  |  | (0.035) |  |  |  |  | (0.018) |  |  |  |
| Strip tillage | 0.460 | 0.032 | 179 | -0.021 |  | 0.563 | 0.131** | 168 | 0.036 |  |
|  |  | (0.113) |  |  |  |  | (0.042) |  |  |  |
| Pit planting | 0.798 | -0.033 | 179 | 0.032 |  | 0.798 | 0.126* | 168 | 0.069 |  |
|  |  | (0.135) |  |  |  |  | (0.054) |  |  |  |
| Contour farming | 0.524 | 0.051 | 179 | -0.006 |  | 0.516 | 0.207* | 168 | 0.078 |  |
|  |  | (0.150) |  |  |  |  | (0.097) |  |  |  |
| Crop rotation | 0.540 | -0.073 | 179 | -0.016 |  | 0.595 | 0.096 | 168 | 0.040 |  |
|  |  | (0.059) |  |  |  |  | (0.048) |  |  |  |
| Row planting | 0.476 | -0.127 | 179 | 0.038 |  | 0.143 | 0.080 | 168 | -0.004 |  |
|  |  | (0.123) |  |  |  |  | (0.113) |  |  |  |
| *See source and notes of S2a Table.* | | | | | | | | | |  |

| Table H. Effect of SLM training intervention (basic and gendered treatments) on contact farmers' SLM adoption. | | | | | | | | | | | |
| --- | --- | --- | --- | --- | --- | --- | --- | --- | --- | --- | --- |
|  | Midline | | | | |  | Endline | | | | |
|  | Ctrl. | Basic treatment | Gendered treatment | N | Adj. |  | Ctrl. | Basic treatment | Gendered treatment | N | Adj. |
|  | Mean |  |  |  | R2 |  | Mean |  |  |  | R2 |
| Mulching | 0.929 | 0.019 | 0.042 | 179 | -0.032 |  | 0.929 | -0.035 | 0.076 | 168 | 0.075 |
|  |  | (0.030) | (0.081) |  |  |  |  | (0.051) | (0.043) |  |  |
| Strip tillage | 0.619 | 0.124 | 0.200 | 179 | 0.036 |  | 0.476 | 0.090 | 0.257** | 168 | 0.066 |
|  |  | (0.100) | (0.114) |  |  |  |  | (0.097) | (0.058) |  |  |
| Pit planting | 0.643 | 0.234*** | 0.273** | 179 | 0.021 |  | 0.476 | 0.004 | 0.143* | 168 | -0.024 |
|  |  | (0.042) | (0.064) |  |  |  |  | (0.062) | (0.060) |  |  |
| Contour farming | 0.405 | 0.140 | 0.217 | 179 | -0.006 |  | 0.048 | 0.098 | 0.133** | 168 | -0.011 |
|  |  | (0.132) | (0.167) |  |  |  |  | (0.054) | (0.035) |  |  |
| Crop rotation | 0.905 | 0.051 | 0.044 | 179 | -0.005 |  | 0.548 | 0.095 | 0.091 | 168 | 0.049 |
|  |  | (0.052) | (0.076) |  |  |  |  | (0.066) | (0.112) |  |  |
| Row planting | 0.524 | 0.009 | 0.095 | 179 | 0.018 |  | 0.357 | 0.142 | 0.140 | 168 | 0.004 |
|  |  | (0.074) | (0.101) |  |  |  |  | (0.086) | (0.102) |  |  |
| *See source and notes of S2a Table.* | | | | | | | | | | | |

| Table I. Effect of SLM training intervention (any treatment) on contact farmers' SLM adoption. | | | | | | | | | |
| --- | --- | --- | --- | --- | --- | --- | --- | --- | --- |
| Adoption | Midline | | | | c | Endline | | | |
|  | Ctrl. | Any treatment | N | Adj. |  | Ctrl. | Any treatment | N | Adj. |
|  | Mean |  |  | R2 |  | Mean |  |  | R2 |
| Mulching | 0.929 | 0.031 | 179 | -0.028 |  | 0.929 | 0.018 | 168 | 0.037 |
|  |  | (0.054) |  |  |  |  | (0.039) |  |  |
| Strip tillage | 0.619 | 0.163 | 179 | 0.036 |  | 0.476 | 0.169* | 168 | 0.050 |
|  |  | (0.099) |  |  |  |  | (0.075) |  |  |
| Pit planting | 0.643 | 0.254*** | 179 | 0.026 |  | 0.476 | 0.070 | 168 | -0.033 |
|  |  | (0.039) |  |  |  |  | (0.042) |  |  |
| Contour farming | 0.405 | 0.179 | 179 | -0.005 |  | 0.048 | 0.115* | 168 | -0.007 |
|  |  | (0.141) |  |  |  |  | (0.045) |  |  |
| Crop rotation | 0.905 | 0.048 | 179 | 0.001 |  | 0.548 | 0.093 | 168 | 0.055 |
|  |  | (0.054) |  |  |  |  | (0.085) |  |  |
| Row planting | 0.524 | 0.053 | 179 | 0.017 |  | 0.357 | 0.141 | 168 | 0.010 |
|  |  | (0.087) |  |  |  |  | (0.088) |  |  |
| *See source and notes of S2a Table.* | | | | | | | | | |

| Table J. Effect of SLM training intervention (basic and gendered treatments) on farmers. | | | | | | | | | | | | |
| --- | --- | --- | --- | --- | --- | --- | --- | --- | --- | --- | --- | --- |
|  |  | Midline | | | | |  | Endline | | | | |
|  |  | Ctrl. | Basic treatment | Gendered treatment | N | Adj. |  | Ctrl. | Basic treatment | Gendered treatment | N | Adj. |
|  |  | Mean |  |  |  | R2 |  | Mean |  |  |  | R2 |
| # of techniques known by name | Women | 2.377 | 0.083 | 0.157 | 3,423 | 0.025 |  | 2.486 | -0.206 | 0.141 | 2,951 | 0.018 |
|  |  |  | (0.150) | (0.148) |  |  |  |  | (0.211) | (0.198) |  |  |
|  | Men | 2.652 | 0.074 | 0.055 | 2,461 | 0.017 |  | 2.941 | -0.351* | -0.071 | 2,120 | 0.023 |
|  |  |  | (0.159) | (0.162) |  |  |  |  | (0.210) | (0.195) |  |  |
| Knowledge score | Women | 0.339 | 0.000 | 0.020 | 3,423 | 0.015 |  | 0.410 | -0.023 | -0.003 | 2,951 | 0.017 |
|  |  |  | (0.015) | (0.015) |  |  |  |  | (0.020) | (0.020) |  |  |
|  | Men | 0.358 | 0.010 | 0.021 | 2,461 | 0.027 |  | 0.449 | -0.025 | -0.008 | 2,120 | 0.020 |
|  |  |  | (0.016) | (0.017) |  |  |  |  | (0.017) | (0.018) |  |  |
| # of techniques adopted | Women | 1.415 | -0.123 | -0.046 | 3,423 | 0.006 |  | 1.757 | -0.001 | 0.128 | 2,951 | 0.006 |
|  |  |  | (0.092) | (0.091) |  |  |  |  | (0.115) | (0.119) |  |  |
|  | Men | 1.560 | -0.071 | -0.074 | 2,461 | 0.012 |  | 2.044 | -0.114 | 0.011 | 2,120 | 0.010 |
|  |  |  | (0.100) | (0.093) |  |  |  |  | (0.127) | (0.132) |  |  |
| *Source: Mozambique Household Survey, 2012, 2013.* Notes: SLM = sustainable land management. Regressions include the following variables: a constant, age, completed at least primary school dummy, single dummy, widow dummy, number of children, total landholdings, the number of rooms in the household, the number of hours worked by the CF at baseline, district indicators, and incentive treatment indicators. Standard errors clustered at the community level in parentheses. ***, **, and * indicate significance at the 1, 5, and 10 percent critical level. | | | | | | | | | | | | |

| Table K. Effect of SLM training intervention (any treatment) on farmers. | | | | | | | | | | |
| --- | --- | --- | --- | --- | --- | --- | --- | --- | --- | --- |
|  |  | Midline | | | | . | Endline | | | |
|  |  | Ctrl. | Any treatment | N | Adj. |  | Ctrl. | Any treatment | N | Adj. |
|  |  | Mean |  |  | R2 |  | Mean |  |  | R2 |
| Knew at least one technique by name | Women | 0.978 | -0.014 | 3,423 | 0.003 |  | 0.947 | -0.003 | 2,951 | 0.003 |
|  |  |  | (0.015) |  |  |  |  | (0.019) |  |  |
|  | Men | 0.981 | 0.005 | 2,461 | 0.006 |  | 0.962 | -0.001 | 2,120 | 0.000 |
|  |  |  | (0.010) |  |  |  |  | (0.015) |  |  |
| # of techniques known by name | Women | 2.377 | 0.120 | 3,423 | 0.024 |  | 2.486 | -0.038 | 2,951 | 0.008 |
|  |  |  | (0.139) |  |  |  |  | (0.192) |  |  |
|  | Men | 2.652 | 0.064 | 2,461 | 0.017 |  | 2.941 | -0.209 | 2,120 | 0.017 |
|  |  |  | (0.149) |  |  |  |  | (0.192) |  |  |
| Knowledge score | Women | 0.339 | 0.010 | 3,423 | 0.010 |  | 0.410 | -0.013 | 2,951 | 0.015 |
|  |  |  | (0.014) |  |  |  |  | (0.019) |  |  |
|  | Men | 0.358 | 0.016 | 2,461 | 0.026 |  | 0.449 | -0.016 | 2,120 | 0.018 |
|  |  |  | (0.016) |  |  |  |  | (0.016) |  |  |
| Adopted at least one technique | Women | 0.876 | -0.036 | 3,423 | 0.004 |  | 0.905 | 0.008 | 2,951 | 0.006 |
|  |  |  | (0.031) |  |  |  |  | (0.022) |  |  |
|  | Men | 0.903 | -0.001 | 2,461 | 0.010 |  | 0.941 | -0.016 | 2,120 | 0.001 |
|  |  |  | (0.026) |  |  |  |  | (0.016) |  |  |
| # of techniques adopted | Women | 1.415 | -0.085 | 3,423 | 0.005 |  | 1.757 | 0.061 | 2,951 | 0.003 |
|  |  |  | (0.085) |  |  |  |  | (0.110) |  |  |
|  | Men | 1.560 | -0.073 | 2,461 | 0.013 |  | 2.044 | -0.050 | 2,120 | 0.008 |
|  |  |  | (0.090) |  |  |  |  | (0.123) |  |  |
| *See source and notes of S6a Table.* | | | | | | | | | | |

| Table L. Effect of SLM training intervention (any treatment) on farmers' SLM awareness. | | | | | | | | | | |
| --- | --- | --- | --- | --- | --- | --- | --- | --- | --- | --- |
|  |  | Midline | | | |  | Endline | | | |
|  |  | Ctrl. | Any treatment | N | Adj. |  | Ctrl. | Any treatment | N | Adj. |
|  |  | Mean |  |  | R2 |  | Mean |  |  | R2 |
| Mulching | Women | 0.418 | -0.012 | 3,423 | 0.038 |  | 0.497 | -0.014 | 2,951 | 0.022 |
|  |  |  | (0.058) |  |  |  |  | (0.060) |  |  |
|  | Men | 0.461 | 0.008 | 2,461 | 0.036 |  | 0.630 | -0.064 | 2,120 | 0.018 |
|  |  |  | (0.058) |  |  |  |  | (0.056) |  |  |
| Strip tillage | Women | 0.236 | -0.019 | 3,423 | 0.110 |  | 0.234 | -0.025 | 2,951 | 0.003 |
|  |  |  | (0.047) |  |  |  |  | (0.045) |  |  |
|  | Men | 0.259 | -0.046 | 2,461 | 0.073 |  | 0.269 | -0.017 | 2,120 | 0.006 |
|  |  |  | (0.052) |  |  |  |  | (0.050) |  |  |
| Pit planting | Women | 0.139 | 0.075** | 3,423 | 0.017 |  | 0.172 | 0.005 | 2,951 | 0.009 |
|  |  |  | (0.031) |  |  |  |  | (0.034) |  |  |
|  | Men | 0.163 | 0.103*** | 2,461 | 0.017 |  | 0.221 | -0.002 | 2,120 | 0.006 |
|  |  |  | (0.037) |  |  |  |  | (0.043) |  |  |
| Contour farming | Women | 0.054 | 0.005 | 3,423 | 0.033 |  | 0.043 | -0.031** | 2,951 | 0.005 |
|  |  |  | (0.016) |  |  |  |  | (0.014) |  |  |
|  | Men | 0.072 | 0.004 | 2,461 | 0.033 |  | 0.048 | -0.011 | 2,120 | 0.001 |
|  |  |  | (0.018) |  |  |  |  | (0.018) |  |  |
| Crop rotation | Women | 0.350 | 0.061* | 3,423 | 0.013 |  | 0.391 | 0.014 | 2,951 | 0.006 |
|  |  |  | (0.035) |  |  |  |  | (0.055) |  |  |
|  | Men | 0.440 | 0.055 | 2,461 | 0.017 |  | 0.450 | -0.009 | 2,120 | 0.007 |
|  |  |  | (0.038) |  |  |  |  | (0.056) |  |  |
| Row planting | Women | 0.128 | -0.003 | 3,423 | 0.015 |  | 0.100 | -0.012 | 2,951 | 0.015 |
|  |  |  | (0.026) |  |  |  |  | (0.034) |  |  |
|  | Men | 0.135 | -0.010 | 2,461 | 0.017 |  | 0.139 | -0.056 | 2,120 | 0.019 |
|  |  |  | (0.033) |  |  |  |  | (0.036) |  |  |
| *See source and notes of S6a Table.* The corresponding results on the separate (i.e. gendered and basic) treatment effects are captured in [28]. | | | | | | | | | | |

| Table M. Effect of SLM training intervention (any treatment) on farmers' SLM knowledge. | | | | | | | | | | |
| --- | --- | --- | --- | --- | --- | --- | --- | --- | --- | --- |
|  |  | Midline | | | |  | Endline | | | |
|  |  | Ctrl. | Any treatment | N | Adj. |  | Ctrl. | Any treatment | N | Adj. |
|  |  | Mean |  |  | R2 |  | Mean |  |  | R2 |
| Mulching | Women | 0.409 | 0.025 | 3,423 | 0.030 |  | 0.606 | -0.018 | 2,951 | 0.014 |
|  |  |  | (0.041) |  |  |  |  | (0.038) |  |  |
|  | Men | 0.440 | 0.051 | 2,461 | 0.039 |  | 0.691 | -0.041 | 2,120 | 0.016 |
|  |  |  | (0.040) |  |  |  |  | (0.034) |  |  |
| Strip tillage | Women | 0.192 | 0.017 | 3,423 | 0.003 |  | 0.304 | -0.015 | 2,951 | 0.001 |
|  |  |  | (0.026) |  |  |  |  | (0.027) |  |  |
|  | Men | 0.218 | -0.004 | 2,461 | 0.004 |  | 0.360 | -0.032 | 2,120 | 0.007 |
|  |  |  | (0.027) |  |  |  |  | (0.028) |  |  |
| Pit planting | Women | 0.253 | 0.054 | 3,423 | 0.005 |  | 0.377 | -0.013 | 2,951 | 0.008 |
|  |  |  | (0.036) |  |  |  |  | (0.044) |  |  |
|  | Men | 0.275 | 0.080* | 2,461 | 0.016 |  | 0.396 | 0.016 | 2,120 | 0.012 |
|  |  |  | (0.040) |  |  |  |  | (0.044) |  |  |
| Contour farming | Women | 0.114 | -0.007 | 3,423 | 0.011 |  | 0.184 | -0.050* | 2,951 | 0.007 |
|  |  |  | (0.019) |  |  |  |  | (0.028) |  |  |
|  | Men | 0.136 | -0.004 | 2,461 | 0.018 |  | 0.205 | -0.038 | 2,120 | 0.015 |
|  |  |  | (0.022) |  |  |  |  | (0.028) |  |  |
| Crop rotation | Women | 0.282 | -0.012 | 3,423 | 0.009 |  | 0.397 | -0.027 | 2,951 | 0.009 |
|  |  |  | (0.020) |  |  |  |  | (0.035) |  |  |
|  | Men | 0.311 | -0.014 | 2,461 | 0.003 |  | 0.450 | -0.038 | 2,120 | 0.012 |
|  |  |  | (0.021) |  |  |  |  | (0.034) |  |  |
| Row planting | Women | 0.333 | -0.028 | 3,423 | 0.006 |  | 0.032 | 0.000 | 2,951 | 0.000 |
|  |  |  | (0.033) |  |  |  |  | (0.017) |  |  |
|  | Men | 0.324 | 0.002 | 2,461 | 0.004 |  | 0.048 | -0.010 | 2,120 | 0.002 |
|  |  |  | (0.032) |  |  |  |  | (0.019) |  |  |
| *See source and notes of S6a Table.* The corresponding results on the separate (i.e. gendered and basic) treatment effects are captured in [28]. | | | | | | | | | | |

| Table N. Effect of SLM training intervention (any treatment) on farmers' SLM adoption. | | | | | | | | | | |
| --- | --- | --- | --- | --- | --- | --- | --- | --- | --- | --- |
|  |  | Midline | | | |  | Endline | | | |
|  |  | Ctrl. | Any treatment | N | Adj. | d | Ctrl. | Any treatment | N | Adj. |
|  |  | Mean |  |  | R2 |  | Mean |  |  | R2 |
| Mulching | Women | 0.248 | -0.058 | 3,423 | 0.016 |  | 0.390 | 0.022 | 2,951 | 0.018 |
|  |  |  | (0.047) |  |  |  |  | (0.052) |  |  |
|  | Men | 0.248 | -0.018 | 2,461 | 0.012 |  | 0.512 | -0.035 | 2,120 | 0.012 |
|  |  |  | (0.047) |  |  |  |  | (0.056) |  |  |
| Strip tillage | Women | 0.153 | -0.042 | 3,423 | 0.065 |  | 0.200 | -0.024 | 2,951 | 0.003 |
|  |  |  | (0.042) |  |  |  |  | (0.038) |  |  |
|  | Men | 0.161 | -0.039 | 2,461 | 0.057 |  | 0.225 | -0.023 | 2,120 | 0.007 |
|  |  |  | (0.043) |  |  |  |  | (0.045) |  |  |
| Pit planting | Women | 0.041 | 0.019 | 3,423 | 0.004 |  | 0.084 | 0.038 | 2,951 | 0.006 |
|  |  |  | (0.019) |  |  |  |  | (0.025) |  |  |
|  | Men | 0.039 | 0.052** | 2,461 | 0.013 |  | 0.139 | -0.002 | 2,120 | 0.002 |
|  |  |  | (0.021) |  |  |  |  | (0.031) |  |  |
| Contour farming | Women | 0.000 | 0.002 | 3,423 | 0.001 |  | 0.007 | -0.007 | 2,951 | 0.002 |
|  |  |  | (0.002) |  |  |  |  | (0.005) |  |  |
|  | Men | 0.000 | 0.003 | 2,461 | -0.001 |  | 0.017 | -0.011 | 2,120 | 0.004 |
|  |  |  | (0.002) |  |  |  |  | (0.011) |  |  |
| Crop rotation | Women | 0.131 | 0.008 | 3,423 | 0.003 |  | 0.151 | 0.042 | 2,951 | 0.005 |
|  |  |  | (0.022) |  |  |  |  | (0.027) |  |  |
|  | Men | 0.182 | -0.013 | 2,461 | 0.005 |  | 0.160 | 0.036 | 2,120 | -0.001 |
|  |  |  | (0.025) |  |  |  |  | (0.031) |  |  |
| Row planting | Women | 0.073 | 0.004 | 3,423 | 0.019 |  | 0.061 | -0.006 | 2,951 | 0.008 |
|  |  |  | (0.021) |  |  |  |  | (0.022) |  |  |
|  | Men | 0.093 | -0.018 | 2,461 | 0.019 |  | 0.095 | -0.036 | 2,120 | 0.014 |
|  |  |  | (0.025) |  |  |  |  | (0.028) |  |  |
| *See source and notes of S6a Table.* The corresponding results on the separate (i.e. gendered and basic) treatment effects are captured in [28]. | | | | | | | | | | |

| Table O. Effect of SLM training intervention (basic and gendered treatments) on pooled farmers. | | | | | | | | | | | |
| --- | --- | --- | --- | --- | --- | --- | --- | --- | --- | --- | --- |
|  | Midline | | | | |  | Endline | | | | |
|  | Ctrl. | Basic treatment | Gendered treatment | N | Adj. |  | Ctrl. | Basic treatment | Gendered treatment | N | Adj. |
|  | Mean |  |  |  | R2 |  | Mean |  |  |  | R2 |
| Knew at least one technique by name | 0.979 | -0.011 | 0.000 | 5,884 | 0.006 |  | 0.953 | -0.005 | -0.001 | 5,071 | 0.002 |
|  |  | (0.013) | (0.011) |  |  |  |  | (0.017) | (0.017) |  |  |
| # of techniques known by name | 2.491 | 0.081 | 0.115 | 5,884 | 0.032 |  | 2.673 | -0.272 | 0.048 | 5,071 | 0.033 |
|  |  | (0.147) | (0.147) |  |  |  |  | (0.204) | (0.190) |  |  |
| Knowledge score | 0.346 | 0.005 | 0.021 | 5,884 | 0.034 |  | 0.426 | -0.024 | -0.005 | 5,071 | 0.028 |
|  |  | (0.015) | (0.015) |  |  |  |  | (0.018) | (0.019) |  |  |
| Adopted at least one technique | 0.887 | -0.026 | -0.016 | 5,884 | 0.007 |  | 0.920 | 0.005 | -0.012 | 5,071 | 0.005 |
|  |  | (0.030) | (0.028) |  |  |  |  | (0.019) | (0.018) |  |  |
| # of techniques adopted | 1.475 | -0.101 | -0.057 | 5,884 | 0.014 |  | 1.875 | -0.053 | 0.074 | 5,071 | 0.018 |
|  |  | (0.091) | (0.087) |  |  |  |  | (0.116) | (0.120) |  |  |
| *Source: Mozambique Household Survey, 2012, 2013.* Notes: SLM = sustainable land management. Regressions include the following variables: a constant, male dummy, age, completed at least primary school dummy, single dummy, widow dummy, number of children, total landholdings, the number of rooms in the household, the number of hours worked by the CF at baseline, district indicators, and incentive treatment indicators. Standard errors clustered at the community level in parentheses. ***, **, and * indicate significance at the 1, 5, and 10 percent critical level. | | | | | | | | | | | |

| Table P. Effect of SLM training intervention (any treatment) on pooled farmers. | | | | | | | | | |
| --- | --- | --- | --- | --- | --- | --- | --- | --- | --- |
|  | Midline | | | |  | Endline | | | |
|  | Ctrl. | Any treatment | N | Adj. |  | Ctrl. | Any treatment | N | Adj. |
|  | Mean |  |  | R2 |  | Mean |  |  | R2 |
| Knew at least one technique by name | 0.979 | -0.006 | 5,884 | 0.006 |  | 0.953 | -0.003 | 5,071 | 0.003 |
|  |  | (0.011) |  |  |  |  | (0.016) |  |  |
| # of techniques known by name | 2.491 | 0.098 | 5,884 | 0.032 |  | 2.673 | -0.115 | 5,071 | 0.025 |
|  |  | (0.137) |  |  |  |  | (0.185) |  |  |
| Knowledge score | 0.346 | 0.013 | 5,884 | 0.031 |  | 0.426 | -0.014 | 5,071 | 0.025 |
|  |  | (0.014) |  |  |  |  | (0.017) |  |  |
| Adopted at least one technique | 0.887 | -0.021 | 5,884 | 0.007 |  | 0.920 | -0.003 | 5,071 | 0.005 |
|  |  | (0.027) |  |  |  |  | (0.017) |  |  |
| # of techniques adopted | 1.475 | -0.079 | 5,884 | 0.014 |  | 1.875 | 0.010 | 5,071 | 0.015 |
|  |  | (0.083) |  |  |  |  | (0.111) |  |  |
| *See source and notes of S10a Table.* | | | | | | | | | |

| Table Q. Effect of SLM training intervention (basic and gendered treatments) on pooled farmers' SLM awareness. | | | | | | | | | | | | | | |
| --- | --- | --- | --- | --- | --- | --- | --- | --- | --- | --- | --- | --- | --- | --- |
|  | Midline | | | | | |  | | Endline | | | | | |
|  | Ctrl. | Basic treatment | Gendered treatment | N | Adj. |  | | Ctrl. | | Basic treatment | Gendered treatment | N | Adj. |  |
|  | Mean |  |  |  | R2 |  | | Mean | |  |  |  | R2 |  |
| Mulching | 0.436 | -0.019 | 0.012 | 5,884 | 0.040 |  | | 0.552 | | -0.081 | 0.015 | 5,071 | 0.037 |  |
|  |  | (0.060) | (0.060) |  |  |  | |  | | (0.060) | (0.059) |  |  |  |
| Strip tillage | 0.245 | -0.034 | -0.025 | 5,884 | 0.092 |  | | 0.248 | | -0.073 | 0.029 | 5,071 | 0.017 |  |
|  |  | (0.051) | (0.051) |  |  |  | |  | | (0.045) | (0.049) |  |  |  |
| Pit planting | 0.149 | 0.077** | 0.098*** | 5,884 | 0.023 |  | | 0.192 | | -0.017 | 0.018 | 5,071 | 0.013 |  |
|  |  | (0.036) | (0.034) |  |  |  | |  | | (0.041) | (0.036) |  |  |  |
| Contour farming | 0.061 | 0.009 | 0.001 | 5,884 | 0.038 |  | | 0.045 | | -0.028* | -0.018 | 5,071 | 0.006 |  |
|  |  | (0.016) | (0.016) |  |  |  | |  | | (0.015) | (0.015) |  |  |  |
| Crop rotation | 0.387 | 0.070* | 0.046 | 5,884 | 0.028 |  | | 0.415 | | -0.017 | 0.021 | 5,071 | 0.009 |  |
|  |  | (0.037) | (0.035) |  |  |  | |  | | (0.057) | (0.058) |  |  |  |
| Row planting | 0.131 | -0.005 | -0.007 | 5,884 | 0.017 |  | | 0.116 | | -0.049 | -0.011 | 5,071 | 0.020 |  |
|  |  | (0.029) | (0.031) |  |  |  | |  | | (0.035) | (0.036) |  |  |  |
| *See source and notes of S10a Table.* | | | | | | | | | | | | | |  |

| Table R. Effect of SLM training intervention (any treatment) on pooled farmers' SLM awareness. | | | | | | | | | | |
| --- | --- | --- | --- | --- | --- | --- | --- | --- | --- | --- |
|  | Midline | | | |  | Endline | | | | |
|  | Ctrl. | Any treatment | N | Adj. |  | Ctrl. | Any treatment | N | Adj. |  |
|  | Mean |  |  | R2 |  | Mean |  |  | R2 |  |
| Mulching | 0.436 | -0.004 | 5,884 | 0.040 |  | 0.552 | -0.034 | 5,071 | 0.029 |  |
|  |  | (0.057) |  |  |  |  | (0.056) |  |  |  |
| Strip tillage | 0.245 | -0.029 | 5,884 | 0.093 |  | 0.248 | -0.023 | 5,071 | 0.005 |  |
|  |  | (0.048) |  |  |  |  | (0.045) |  |  |  |
| Pit planting | 0.149 | 0.088*** | 5,884 | 0.023 |  | 0.192 | 0.000 | 5,071 | 0.011 |  |
|  |  | (0.032) |  |  |  |  | (0.036) |  |  |  |
| Contour farming | 0.061 | 0.005 | 5,884 | 0.037 |  | 0.045 | -0.024* | 5,071 | 0.006 |  |
|  |  | (0.015) |  |  |  |  | (0.014) |  |  |  |
| Crop rotation | 0.387 | 0.058* | 5,884 | 0.027 |  | 0.415 | 0.002 | 5,071 | 0.008 |  |
|  |  | (0.034) |  |  |  |  | (0.054) |  |  |  |
| Row planting | 0.131 | -0.006 | 5,884 | 0.017 |  | 0.116 | -0.030 | 5,071 | 0.017 |  |
|  |  | (0.028) |  |  |  |  | (0.034) |  |  |  |
| *See source and notes of S10a Table.* | | | | | | | | | |  |

| Table S. Effect of SLM training intervention (basic and gendered treatments) on pooled farmers' SLM knowledge. | | | | | | | | | | | | | |
| --- | --- | --- | --- | --- | --- | --- | --- | --- | --- | --- | --- | --- | --- |
|  | Midline | | | | | |  | | Endline | | | | |
|  | Ctrl. | Basic treatment | Gendered treatment | N | Adj. |  | | Ctrl. | | Basic treatment | Gendered treatment | N | Adj. |
|  | Mean |  |  |  | R2 |  | | Mean | |  |  |  | R2 |
| Mulching | 0.422 | 0.020 | 0.052 | 5,884 | 0.041 |  | | 0.641 | | -0.056 | 0.004 | 5,071 | 0.026 |
|  |  | (0.042) | (0.043) |  |  |  | |  | | (0.037) | (0.035) |  |  |
| Strip tillage | 0.203 | 0.000 | 0.017 | 5,884 | 0.007 |  | | 0.327 | | -0.034 | -0.009 | 5,071 | 0.009 |
|  |  | (0.027) | (0.028) |  |  |  | |  | | (0.027) | (0.029) |  |  |
| Pit planting | 0.262 | 0.041 | 0.088** | 5,884 | 0.017 |  | | 0.385 | | -0.018 | 0.013 | 5,071 | 0.012 |
|  |  | (0.040) | (0.039) |  |  |  | |  | | (0.045) | (0.044) |  |  |
| Contour farming | 0.123 | -0.015 | 0.003 | 5,884 | 0.019 |  | | 0.193 | | -0.063** | -0.028 | 5,071 | 0.016 |
|  |  | (0.021) | (0.021) |  |  |  | |  | | (0.028) | (0.029) |  |  |
| Crop rotation | 0.294 | -0.015 | -0.011 | 5,884 | 0.010 |  | | 0.419 | | -0.041 | -0.022 | 5,071 | 0.013 |
|  |  | (0.020) | (0.021) |  |  |  | |  | | (0.036) | (0.037) |  |  |
| Row planting | 0.329 | -0.014 | -0.017 | 5,884 | 0.006 |  | | 0.038 | | -0.006 | -0.003 | 5,071 | 0.001 |
|  |  | (0.032) | (0.031) |  |  |  | |  | | (0.019) | (0.016) |  |  |
| *See source and notes of S10a Table.* | | | | | | | | | | | | | |

| Table T. Effect of SLM training intervention (any treatment) on pooled farmers' SLM knowledge. | | | | | | | | | | |
| --- | --- | --- | --- | --- | --- | --- | --- | --- | --- | --- |
|  | Midline | | | |  | Endline | | | | |
|  | Ctrl. | Any treatment | N | Adj. |  | Ctrl. | Any treatment | N | Adj. |  |
|  | Mean |  |  | R2 |  | Mean |  |  | R2 |  |
| Mulching | 0.422 | 0.036 | 5,884 | 0.040 |  | 0.641 | -0.026 | 5,071 | 0.021 |  |
|  |  | (0.040) |  |  |  |  | (0.033) |  |  |  |
| Strip tillage | 0.203 | 0.008 | 5,884 | 0.006 |  | 0.327 | -0.022 | 5,071 | 0.008 |  |
|  |  | (0.026) |  |  |  |  | (0.026) |  |  |  |
| Pit planting | 0.262 | 0.065* | 5,884 | 0.015 |  | 0.385 | -0.003 | 5,071 | 0.011 |  |
|  |  | (0.036) |  |  |  |  | (0.042) |  |  |  |
| Contour farming | 0.123 | -0.006 | 5,884 | 0.018 |  | 0.193 | -0.046* | 5,071 | 0.013 |  |
|  |  | (0.019) |  |  |  |  | (0.027) |  |  |  |
| Crop rotation | 0.294 | -0.013 | 5,884 | 0.010 |  | 0.419 | -0.032 | 5,071 | 0.012 |  |
|  |  | (0.019) |  |  |  |  | (0.033) |  |  |  |
| Row planting | 0.329 | -0.015 | 5,884 | 0.007 |  | 0.038 | -0.004 | 5,071 | 0.002 |  |
|  |  | (0.029) |  |  |  |  | (0.017) |  |  |  |
| *See source and notes of S10a Table.* | | | | | | | | | |  |

| Table U. Effect of SLM training intervention (basic and gendered treatments) on pooled farmers' SLM adoption. | | | | | | | | | | | |
| --- | --- | --- | --- | --- | --- | --- | --- | --- | --- | --- | --- |
|  | Midline | | | | |  | Endline | | | | |
|  | Ctrl. | Basic treatment | Gendered treatment | N | Adj. |  | Ctrl. | Basic treatment | Gendered treatment | N | Adj. |
|  | Mean |  |  |  | R2 |  | Mean |  |  |  | R2 |
| Mulching | 0.248 | -0.066 | -0.015 | 5,884 | 0.018 |  | 0.440 | -0.026 | 0.024 | 5,071 | 0.025 |
|  |  | (0.047) | (0.051) |  |  |  |  | (0.055) | (0.056) |  |  |
| Strip tillage | 0.156 | -0.039 | -0.041 | 5,884 | 0.062 |  | 0.210 | -0.064* | 0.015 | 5,071 | 0.013 |
|  |  | (0.046) | (0.043) |  |  |  |  | (0.039) | (0.043) |  |  |
| Pit planting | 0.040 | 0.035* | 0.033* | 5,884 | 0.009 |  | 0.107 | 0.016 | 0.025 | 5,071 | 0.009 |
|  |  | (0.020) | (0.019) |  |  |  |  | (0.029) | (0.026) |  |  |
| Contour farming | 0.000 | 0.004* | 0.001 | 5,884 | 0.002 |  | 0.011 | -0.007 | -0.011 | 5,071 | 0.006 |
|  |  | (0.003) | (0.001) |  |  |  |  | (0.008) | (0.007) |  |  |
| Crop rotation | 0.152 | -0.001 | -0.003 | 5,884 | 0.007 |  | 0.155 | 0.034 | 0.044 | 5,071 | 0.003 |
|  |  | (0.023) | (0.023) |  |  |  |  | (0.027) | (0.030) |  |  |
| Row planting | 0.081 | -0.007 | -0.003 | 5,884 | 0.020 |  | 0.075 | -0.028 | -0.009 | 5,071 | 0.013 |
|  |  | (0.023) | (0.024) |  |  |  |  | (0.023) | (0.025) |  |  |
| *See source and notes of S10a Table.* | | | | | | | | | | | |

| Table V. Effect of SLM training intervention (any treatment) on pooled farmers' SLM adoption. | | | | | | | | | |
| --- | --- | --- | --- | --- | --- | --- | --- | --- | --- |
|  | Midline | | | |  | Endline | | | |
|  | Ctrl. | Any treatment | N | Adj. |  | Ctrl. | Any treatment | N | Adj. |
|  | Mean |  |  | R2 |  | Mean |  |  | R2 |
| Mulching | 0.248 | -0.041 | 5,884 | 0.015 |  | 0.440 | -0.001 | 5,071 | 0.023 |
|  |  | (0.046) |  |  |  |  | (0.052) |  |  |
| Strip tillage | 0.156 | -0.040 | 5,884 | 0.062 |  | 0.210 | -0.025 | 5,071 | 0.005 |
|  |  | (0.041) |  |  |  |  | (0.039) |  |  |
| Pit planting | 0.040 | 0.034* | 5,884 | 0.009 |  | 0.107 | 0.020 | 5,071 | 0.009 |
|  |  | (0.018) |  |  |  |  | (0.026) |  |  |
| Contour farming | 0.000 | 0.003 | 5,884 | 0.001 |  | 0.011 | -0.009 | 5,071 | 0.005 |
|  |  | (0.002) |  |  |  |  | (0.007) |  |  |
| Crop rotation | 0.152 | -0.002 | 5,884 | 0.008 |  | 0.155 | 0.039 | 5,071 | 0.003 |
|  |  | (0.022) |  |  |  |  | (0.026) |  |  |
| Row planting | 0.081 | -0.005 | 5,884 | 0.021 |  | 0.075 | -0.019 | 5,071 | 0.012 |
|  |  | (0.022) |  |  |  |  | (0.023) |  |  |
| *See source and notes of S10a Table.* | | | | | | | | | |

| Table W. Results of cost-effectiveness analysis of land advisory services, treatment based on assignment and compliance. | | | | | | | | | | | | |  |
| --- | --- | --- | --- | --- | --- | --- | --- | --- | --- | --- | --- | --- | --- |
|  | Believes wife should inherit | | | | Believes land and housing tribunal treats cases fairly | | | | Aware of paralegal in village | | | | |
|  | Men | Women | Men | Women | Men | Women | Men | Women | Men | Women | Men | Women | |
| CBLA treatment assignments by village (ITT) | –0.01 | 0.06** |  |  | 0.05 | 0.04 |  |  | 0.12*** | 0.07*** |  |  | |
|  | (0.04) | (0.03) |  |  | (0.04) | (0.03) |  |  | (0.03) | (0.02) |  |  | |
| CBLA actual treatment (TOT) |  |  | –0.01 | 0.07** |  |  | 0.05 | 0.05 |  |  | 0.15*** | 0.08*** | |
|  |  |  | (0.05) | (0.03) |  |  | (0.05) | (0.03) |  |  | (0.04) | (0.02) | |
| R^2^ | 0.05 | 0.03 | 0.05 | 0.02 | 0.03 | 0.05 | 0.03 | 0.05 | 0.04 | 0.03 | 0.01 | 0.03 | |
| N | 638 | 1,241 | 638 | 1,241 | 638 | 1,241 | 638 | 1,241 | 638 | 1,241 | 638 | 1,241 | |
| Bonferroni–Sidak adjusted p-value | 1.00 | 0.16 |  |  | 0.66 | 0.48 |  |  | 0.00 | 0.00 |  |  | |
| Kleibergen–Paap Wald F statistic |  |  | 376.66 | 348.36 |  |  | 376.66 | 348.36 |  |  | 376.66 | 348.36 | |
| Baseline means | 0.68 | 0.63 |  |  | 0.46 | 0.32 |  |  | 0.04 | 0.03 |  |  | |
| *Source: Tanzania Household Survey and Community Survey, 2013, 2014. [29].* Notes: ITT = intent to treat; TOT = treatment on the treated. Standard errors in parentheses. Baseline marital status, education, age, age-squared, land tercile categories, and district variables included as control variables.***, **, and * indicate significance at the 1, 5, and 10 percent critical level. | | | | | | | | | | | | |  |

| (Table W cont’d): | | | | | | | | | | | | | |
| --- | --- | --- | --- | --- | --- | --- | --- | --- | --- | --- | --- | --- | --- |
|  | Answered question correctly about who to approach in unresolved large land conflict | | | | Answered question correctly about recognised son’s entitlement to inheritance | | | | | Answered question correctly about government having the right to expropriate land for public use | | | |
|  | Men | Women | Men | Women | Men | Women | Men | Women | Men | | Women | Men | Women |
| CBLA treatment assignments by village (ITT) | 0.10** | 0.02 |  |  | –0.04 | 0.03 |  |  | 0.17*** | | 0.11** |  |  |
|  | (0.04) | (0.01) |  |  | (0.04) | (0.04) |  |  | (0.06) | | (0.05) |  |  |
| CBLA actual treatment (TOT) |  |  | 0.12** | 0.02 |  |  | –0.05 | 0.04 |  | |  | 0.20*** | 0.13** |
|  |  |  | (0.05) | (0.02) |  |  | (0.05) | (0.05) |  | |  | (0.08) | (0.06) |
| R^2^ | 0.02 | 0.01 | 0.02 | 0.01 | 0.04 | 0.01 | 0.04 | 0.01 | 0.02 | | 0.01 | 0.01 | 0.01 |
| N | 638 | 1,241 | 638 | 1,241 | 618 | 1,219 | 618 | 1,219 | 618 | | 1,219 | 618 | 1,219 |
| Bonferroni–Sidak adjusted p-value | 0.05 | 0.61 |  |  | 0.85 | 0.92 |  |  | 0.03 | | 0.14 |  |  |
| Kleibergen–Paap Wald F statistic |  |  | 376.66 | 348.36 |  |  | 350.28 | 364.07 |  | |  | 350.28 | 364.07 |
| Baseline means | 0.16 | 0.04 |  |  | 0.83 | 0.77 |  |  | 0.62 | | 0.40 |  |  |

| (Table W cont’d): | | | | |  |  |
| --- | --- | --- | --- | --- | --- | --- |
|  | Attended seminar on legal rights in the last 12 months | | | | | |
|  | Men | Women | Men | Women | |  |
| CBLA treatment assignments by village (ITT) | 0.01 | –0.00 |  |  | |  |
|  | (0.01) | (0.00) |  |  | |  |
| CBLA actual treatment (TOT) |  |  | 0.01 | –0.01 | |  |
|  |  |  | (0.01) | (0.01) | |  |
| R2 | 0.02 | 0.02 | 0.02 | 0.01 | |  |
| N | 618 | 1,219 | 618 | 1,219 | |  |
| Bonferroni–Sidak adjusted p-value | 0.92 | 0.76 |  |  | |  |
| Kleibergen–Paap Wald F statistic |  |  | 350.28 | 364.07 | |  |
| Baseline means | 0.02 | 0.01 |  |  | |  |

| Table X. Results of cost-effectiveness analysis of land advisory services, accounting for spillovers. | | | | | | | | |
| --- | --- | --- | --- | --- | --- | --- | --- | --- |
|  | Believes wife should inherit | | Believes land and housing tribunal treats cases fairly | | Aware of paralegal in village | | Answered question correctly about who to approach in unresolved large land conflict | |
|  | Men | Women | Men | Women | Men | Women | Men | Women |
| Spillover | –0.03 | 0.04 | 0.11** | 0.09** | 0.06 | –0.03 | –0.06 | –0.01 |
|  | (0.07) | (0.05) | (0.05) | (0.03) | (0.04) | (0.03) | (0.07) | (0.02) |
| Pure effects (control excludes spillover areas) | –0.02 | 0.07** | 0.08* | 0.06** | 0.14*** | 0.06*** | 0.08** | 0.01 |
|  | (0.04) | (0.03) | (0.04) | (0.03) | (0.04) | (0.02) | (0.04) | (0.01) |
| R2 | 0.05 | 0.03 | 0.04 | 0.06 | 0.04 | 0.03 | 0.02 | 0.01 |
| N | 638 | 1,241 | 638 | 1,241 | 638 | 1,241 | 638 | 1,241 |
| Baseline means | 0.69 | 0.63 | 0.45 | 0.31 | 0.04 | 0.03 | 0.17 | 0.04 |
| *Source: Tanzania Household Survey and Community Survey, 2013, 2014. [29].* Notes: Standard errors in parentheses. Baseline means and standard errors computed using inverse probability weights. Baseline marital status, education, age, age-squared, land tercile categories, and district variables included als control variables. ***, **, and * indicate significance at the 1, 5, and 10 percent critical level. | | | | | | | | |

| (Table X cont’d): | | | | | | | | |  |
| --- | --- | --- | --- | --- | --- | --- | --- | --- | --- |
|  | Answered question correctly about recognised son’s entitlement to inheritance | | | Answered question correctly about government having the right to expropriate land for public use | | Attended seminar on legal rights in the last 12 months | |  |  |
|  | Men | Women | Men | | Women | Men | Women | | |
| Spillover | 0.15** | –0.01 | 0.02 | | 0.04 | 0.01 | 0.02** | | |
|  | (0.06) | (0.07) | (0.11) | | (0.08) | (0.02) | (0.01) | | |
| Pure effects (control excludes spillover areas) | 0.01 | 0.03 | 0.18** | | 0.12** | 0.01 | 0.00 | | |
|  | (0.05) | (0.04) | (0.07) | | (0.06) | (0.01) | (0.00) | | |
| R2 | 0.05 | 0.01 | 0.02 | | 0.01 | 0.02 | 0.02 | | |
| N | 618 | 1,219 | 618 | | 1,219 | 618 | 1,219 | | |
| Baseline means | 0.83 | 0.77 | 0.62 | | 0.39 | 0.02 | 0.01 | | |

**C. Additional descriptive statistics on costs and coverage**

| Table Y. Summary of costs of the agricultural SLM advisory services programme, by year and tier. | | | | | |
| --- | --- | --- | --- | --- | --- |
| Administrative level | 2010 | 2011 | 2012 | 2013 | All years |
| Community | 781 | 2,939 | 8,057 | 1,592 | 13,369 |
| Post | 2,066 | 7,200 | 12,210 | 1,649 | 23,125 |
| District | 10,127 | 14,997 | 14,130 | 6,760 | 46,015 |
| Central | 3,465 | 7,257 | 9,564 | 6,641 | 26,927 |
| All tiers | 16,440 | 32,392 | 43,961 | 16,643 | 109,436 |
| Note: Values in 1,000 Mozambican meticais. Exchange rate prevailing in mid-2013: US$1 = 29.6 MZN. | | | | | |

| Table Z. Coverage under scale-up scenarios of the of agricultural SLM advisory services programme. | | | | | | | | | |
| --- | --- | --- | --- | --- | --- | --- | --- | --- | --- |
| Coverage |  | Sofala  Province | |  | Tete province |  | Zambezia  Province | | All locations |
|  |  | Chemba  district | Maringue  district |  | Mutarara district |  | Mopeia district | Morrumbala district |  |
| *Programme coverage under scenario of scale-up to saturate localities* | | | | | | | | |  |
| Communities under basic treatment | | 129 | 110 |  | 112 |  | 63 | 223 | 637 |
| Communities under gendered treatment | | 129 | 136 |  | 116 |  | 60 | 130 | 571 |
| Individuals exposed to basic treatment | *Men* | 11,412 | 10,725 |  | 42,816 |  | 27,270 | 88,535 | 180,758 |
|  | *Women* | 10,547 | 10,229 |  | 39,296 |  | 24,934 | 80,213 | 165,219 |
|  | *All* | 21,959 | 20,954 |  | 82,112 |  | 52,204 | 168,748 | 345,977 |
| Individuals exposed to gendered treatment | *Men* | 10,757 | 12,648 |  | 42,171 |  | 27,128 | 57,115 | 149,819 |
|  | *Women* | 9,949 | 12,130 |  | 38,853 |  | 24,806 | 51,711 | 137,449 |
|  | *All* | 20,706 | 24,778 |  | 81,024 |  | 51,934 | 108,826 | 287,268 |
| *Programme coverage under scenario of scale-up to saturate posts/districts* | | | | | | | | |  |
| Communities under single treatment | | 129 | 136 |  | 133 |  | 63 | 243 | 704 |
| Individuals exposed to single treatment | *Men* | 11,412 | 15,887 |  | 53,455 |  | 27,966 | 90,844 | 199,564 |
|  | *Women* | 10,547 | 15,220 |  | 49,351 |  | 25,557 | 82,286 | 182,961 |
|  | *All* | 21,959 | 31,108 |  | 102,805 |  | 53,523 | 173,130 | 382,524 |
| Notes: Given that in our project area each treatment arm is present in at least one community of each post, three features hold: for each district (1) the number of posts involving the basic treatment and the gendered treatment is identical to the total number of posts under both scale-up scenarios; (2) in the scale-up to posts/districts scenario, the number of communities and farmers under the basic treatment is equal to the number under gendered treatment; and (3) in the scale-up to posts/districts scenario, the number of localities involving a single treatment arm is identical to the total number of localities. | | | | | | | | | |

**D. Sensitivity analysis on discount rate and capital durability**

| Table Aa. Cost-effectiveness of the gendered treatment of the SLM programme in increasing contact farmers’ SLM knowledge. | | | | | |
| --- | --- | --- | --- | --- | --- |
| Useful life in years: | 5 | |  | 20 | |
| Discount rate: | 1% | 10% |  | 1% | 10% |
| Contour farming | 711.07** | 493.11** |  | 662.98** | 462.13** |
| Strip tillage | 917.62*** | 636.35*** |  | 855.56*** | 596.36*** |
| Pit planting | 1,470.99* | 1,020.10* |  | 1,371.52* | 956.00* |
| Crop rotation | 1,529.37* | 1,060.59* |  | 1,425.94* | 993.94* |
| Mulching | 5,071.06* | 3,516.68* |  | 4,728.12* | 3,295.69* |
| General SLM | 1,736.04** | 1,203.91** |  | 1,618.64** | 1,128.25** |
| Note: SLM = sustainable land management. Values in 1,000 Mozambican meticais. Exchange rate prevailing in mid-2013: US$1 = 29.6 MZN. Cost-effectiveness ratios are based on costs and impact by the time of the endline survey. Full cost-inclusiveness (corresponding to the “central” column in Table 6). Sensitivity analysis considers halving (to 5) and doubling (to 20) the assumed useful life of 10 years of capital items in the Mozambique programme. For these, sensitivity is also reported to variations in the assumed discount rate of 3%, namely one third that (1%) and more than three times that (10%). CERs are only reported for cases with statistically significant underlying effects (see further details in Section 2.6). ***, **, and * indicate statistical significance at the 1, 5, and 10 per cent levels, respectively. | | | | | |

| Table Ab. Cost-effectiveness in increasing awareness of the pit planting technique of SLM. | | | | | | |
| --- | --- | --- | --- | --- | --- | --- |
| Gender | Treatment arm | 5 | |  | 20 | |
|  |  | 1% | 10% |  | 1% | 10% |
| All | Basic tr. | 6.15** | 4.36** |  | 5.78** | 4.11** |
|  | Gendered tr. | 3.62*** | 2.56*** |  | 3.42*** | 2.42*** |
| Men | Basic tr. | 8.81* | 6.24* |  | 8.28* | 5.88* |
|  | Gendered tr. | 6.58* | 4.65* |  | 6.22* | 4.40* |
| Women | Basic tr. | 17.38* | 12.31* |  | 16.33* | 11.60* |
|  | Gendered tr. | 8.00** | 5.65** |  | 7.56** | 5.35** |
| Note: SLM = sustainable land management. Values in 1,000 Mozambican meticais. Exchange rate prevailing in mid-2013: cUS$1 = 29.6 MZN. Cost-effectiveness ratios are based on costs and impact by the time of the midline survey. Coverage based on household size estimation from the household survey. Full cost-inclusiveness (corresponding to the “central” column in Table 8). Sensitivity analysis considers halving (to 5) and doubling (to 20) the assumed useful life of 10 years of capital items in the Mozambique programme. For these, sensitivity is also reported to variations in the assumed discount rate of 3%, namely one third that (1%) and more than three times that (10%). CERs are only reported for cases with statistically significant underlying effects (see further details in Section 2.6). ***, **, and * indicate statistical significance at the 1, 5, and 10 per cent levels, respectively. | | | | | | |

| Table Ac. Cost-effectiveness in increasing adoption of pit planting of SLM, under scale-up scenarios. | | | | | | |
| --- | --- | --- | --- | --- | --- | --- |
| Gender | Treatment arm | 5 | | 20 | | |
|  |  | 1% | 10% |  | 1% | 10% |
| *Scale-up within localities* | |  |  |  |  |  |
| All | Basic tr. | 8.00* | 5.61* |  | 7.62* | 5.35* |
|  | Gendered tr. | 11.45* | 8.01* |  | 10.98* | 7.69* |
| Men | Basic tr. | 9.08** | 6.37** |  | 8.65** | 6.08** |
|  | Gendered tr. | 16.47** | 11.52** |  | 15.78** | 11.06** |
| *Scale-up within posts/districts* | |  |  |  |  |  |
| All | Basic tr. | 7.68* | 5.38* |  | 7.33* | 5.14* |
|  | Gendered tr. | 9.62* | 6.72* |  | 9.24* | 6.46* |
| Men | Basic tr. | 8.73** | 6.12** |  | 8.33** | 5.85** |
|  | Gendered tr. | 13.83** | 9.65** |  | 13.29** | 9.29** |
| Note: Values in 1,000 Mozambican meticais. Exchange rate prevailing in mid-2013: US$1 = 29.6 MZN. Cost-effectiveness ratios are based on costs and impact by the time of the midline survey. Full cost-inclusiveness (corresponding to the “central” column in Table 11). Sensitivity analysis considers halving (to 5) and doubling (to 20) the assumed useful life of 10 years of capital items in the Mozambique programme. For these, sensitivity is also reported to variations in the assumed discount rate of 3%, namely one third that (1%) and more than three times that (10%). CERs are only reported for cases with statistically significant underlying effects (see further details in Section 2.6). ***, **, and * indicate statistical significance at the 1, 5, and 10 per cent levels, respectively. | | | | | | |

| Table Ad. Sensitivity analysis on discount rate and capital durability in paralegal land advisory services programme. | | | | | | | | | | |
| --- | --- | --- | --- | --- | --- | --- | --- | --- | --- | --- |
| Outcome | Discount rate | Useful capital life | Based on treatment assignment | | Based on actual treatment | | Spillover effects | | Pure effects (control excludes spillover areas) | |
|  |  |  | Men | Women | Men | Women | Men | Women | Men | Women |
| Believes wife should inherit | 0.01 | 1 |  | 41.74** |  | 43.58** |  |  |  | 34.97** |
|  |  | 4 |  | 41.38** |  | 43.20** |  |  |  | 34.67** |
|  | 0.10 | 1 |  | 40.37** |  | 42.15** |  |  |  | 33.82** |
|  |  | 4 |  | 40.03** |  | 41.79** |  |  |  | 33.54** |
| Believes land and housing tribunal treats cases fairly | 0.01 | 1 |  |  |  |  | 109.24** | 114.27** | 32.70* | 36.53** |
|  |  | 4 |  |  |  |  | 108.30** | 113.28** | 32.41* | 36.22** |
|  | 0.10 | 1 |  |  |  |  | 105.67** | 110.53** | 31.63* | 35.34** |
|  |  | 4 |  |  |  |  | 104.77** | 109.59** | 31.36* | 35.04** |
| Aware of paralegal in village | 0.01 | 1 | 20.77*** | 33.60*** | 21.93*** | 35.08*** |  |  | 18.07*** | 38.82*** |
|  |  | 4 | 20.59*** | 33.31*** | 21.74*** | 34.78*** |  |  | 17.92*** | 38.48*** |
|  | 0.10 | 1 | 20.09*** | 32.50*** | 21.21*** | 33.94*** |  |  | 17.48*** | 37.55*** |
|  |  | 4 | 19.92*** | 32.23*** | 21.03*** | 33.65*** |  |  | 17.33*** | 37.23*** |
| Answered question correctly about who to approach in unresolved large land conflict | 0.01 | 1 | 25.40** |  | 26.82** |  |  |  | 30.65** |  |
|  |  | 4 | 25.18** |  | 26.59** |  |  |  | 30.38** |  |
|  | 0.10 | 1 | 24.57** |  | 25.95** |  |  |  | 29.65** |  |
|  |  | 4 | 24.36** |  | 25.73** |  |  |  | 29.39** |  |
| Answered question correctly about recognized son’s entitlement to inheritance | 0.01 | 1 |  |  |  |  | 80.42** |  |  |  |
|  |  | 4 |  |  |  |  | 79.73** |  |  |  |
|  | 0.10 | 1 |  |  |  |  | 77.79** |  |  |  |
|  |  | 4 |  |  |  |  | 77.13** |  |  |  |
| Answered question correctly about government having the right to expropriate land for public use | 0.01 | 1 | 14.99*** | 21.52** | 15.73*** | 22.56** |  |  | 14.47** | 19.42** |
|  |  | 4 | 14.86*** | 21.33** | 15.60*** | 22.37** |  |  | 14.34** | 19.26** |
|  | 0.10 | 1 | 14.50*** | 20.81** | 15.22*** | 21.83** |  |  | 13.99** | 18.79** |
|  |  | 4 | 14.38*** | 20.64** | 15.09*** | 21.64** |  |  | 13.87** | 18.63** |
| Attended seminar on legal rights in the last 12 months | 0.01 | 1 |  |  |  |  |  | 493.97** |  |  |
|  |  | 4 |  |  |  |  |  | 489.70** |  |  |
|  | 0.10 | 1 |  |  |  |  |  | 477.81** |  |  |
|  |  | 4 |  |  |  |  |  | 473.75** |  |  |
| Note: Cost-effectiveness ratio values in 1,000 Tanzanian shillings. Exchange rate prevailing in mid-2014: US$1 = 1,626 TZS. The figures in the column “Useful capital life” signify the multipliers used on US government indicators of the length of life of various capital equipment and items. Thus, in rows with the number 1, the US indicators are used in the Tanzania data. In rows with the number 4, the length of equipment life in the data is assumed to be four times that in US accounting standards. CERs are only reported for cases with statistically significant underlying effects (see further details in Section 2.6). ***, **, and * indicate statistical significance at the 1, 5, and 10 per cent levels, respectively. | | | | | | | | | | |
